# Degradation of 2‑Amino-2-methylpropanol and Piperazine at CO_2_ Capture-Relevant Conditions

**DOI:** 10.1021/acs.iecr.5c00527

**Published:** 2025-05-23

**Authors:** Vanja Buvik, Kai Vernstad, Andreas Grimstvedt, Karen K. Høisæter, Solrun J. Vevelstad, Hanna K. Knuutila

**Affiliations:** † 275243 SINTEF Industry, Trondheim 7465, Norway; ‡ Technology Centre Mongstad, Mongstad 5954, Norway; § Department of Chemical Engineering, NTNU, Trondheim 7491, Norway

## Abstract

The degradation pathways of the nonproprietary solvent blend CESAR1, consisting of 3.0 M 2-amino-2-methylpropanol (AMP) and 1.5 M piperazine (PZ), were studied under oxidative and thermal stress separately. Liquid chromatography coupled with mass spectrometry, using analytical standards of known and suggested degradation compounds, allowed for the identification and quantification of known, proposed, and new degradation products in the solvent. A total of 48 different degradation compounds were quantified in the degraded solvent samples. AMP is highly resistant to oxidative stress compared to PZ, and the single amines degrade more slowly alone than together in the CESAR1 blend, which degrades more than twice as fast as PZ. The main products found in the solvent after oxidative CESAR1 degradation were formic acid, 1-piperazinecarboxaldehyde, ethylenediamine, *N*-(2-hydroxy-1,1-dimethylethyl)­glycine, formaldehyde, and piperazinone, while the products of thermal degradation were 4,4-dimethyl-2-oxazolidinone, α,α-dimethyl-1-piperazineethanamine, ammonia, 2-[(2-amino-2-methylpropyl)­amino]-2-methyl-1-propanol, 3,4,4-trimethyl-2-oxazolidinone, and acetone. PZ is more resilient under thermal stress than AMP and CESAR1. Fifteen of the compounds included in this study have not previously been quantified in AMP, PZ, or CESAR1. It was found that nitrosamines and nitramines form readily in CESAR1 under oxidizing conditions, even in the absence of nitrogen oxides in the reaction gas, and that *N*-nitrosopiperazine is one of the ten most abundant degradation species in oxidized CESAR1. At nearly all tested conditions, the total nitrogen concentration of the solvent correlates well with the sum of specific nitrogen-containing compounds, meaning that the most abundant degradation compounds have been identified in this work. At 150 °C, some thermal CESAR1 degradation compounds remain unidentified.

## Graphical abstract

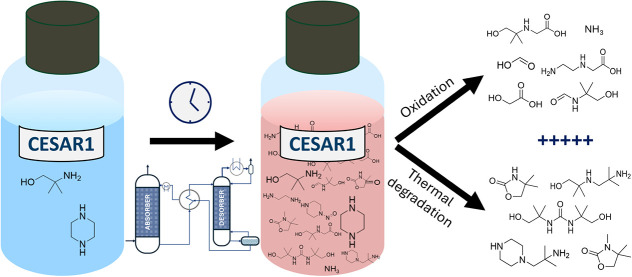

## 1 Introduction

The nonproprietary CESAR1 solvent blend, consisting of 1.5 mol/kg piperazine (PZ) and 3.0 mol/kg 2-amino-2-methylpropanol (AMP),^1,2^ was formulated in the EU project CESAR (CO_2_ Enhanced Separation and Recovery), which took place from 2008 to 2011.^3^ CESAR1 has been widely tested and proven to provide lower energy consumption, better flexibility, and higher chemical stability than many other solvents, notably the former benchmark monoethanolamine (MEA).^4–9^ It has been piloted in many locations, with various flue gases, using various process conditions, and appears to be one of the most promising solvent blends for the future commercial market for CO_2_ capture from industrial sources.^2,7,8,10–19^

Although the solvent stability is high, full-scale and widespread implementation of CESAR1 for post-combustion capture (PCC) of CO_2_ will require a robust assessment of how the solvent will change during long-term operation. Solvent degradation can impact operational costs and cause issues such as corrosion, contamination of the CO_2_ product, and potentially give rise to environmental issues if mitigation technologies used to avoid emissions of volatile compounds are not correctly designed.^4,20,21^ If compounds other than solvent amines are possible process emissions, monitoring and control systems must be adapted accordingly to ensure an environmentally benign process. Knowing how to monitor the solvent and which compounds to expect allows for predictable operation, which in turn reduces both costs and risks associated with the PCC process. Knowledge of solvent degradation chemistry can also support the solvent management strategies to be implemented, such as how and when reclamation should optimally be performed.

Solvent degradation takes place due to many factors in the PCC process, like the presence of oxidizing species, dissolved metals, dispersed particles, and high temperatures.^22–24^ The leading cause of solvent loss and decomposition has been found to be oxidative degradation, mainly caused by oxygen in the flue gas.^25,26^ Other important mechanisms are thermal degradation, caused by the elevated temperatures under which CO_2_ desorption takes place, and nitrosation reactions as a result of NO_*X*_ being present in the flue gas. Solvent degradation is also closely linked to material corrosion, as the concentration of dissolved metals typically increases in pace with the degree of degradation.^22,27^ Degradation compounds are hypothesized to increase the solubility of metals by complexation and/or pH changes, thereby increasing corrosion; however, dissolved metals are also known to catalyze especially the oxidative degradation reactions.^7,27^

Initial degradation studies of solvents for CO_2_ capture are often conducted under either oxidative or thermal stress separately, before being studied under cyclic conditions at a later stage. In this work, we also use thermal and oxidative degradation tests to identify the conditions under which each degradation compound is formed, and thereby suggest degradation mechanisms. The degradation products formed under these conditions are in line with those found in a CESAR1 solvent sample after operation at pilot scale at the Technology Centre Mongstad (TCM).^28^ Much of the available data on degradation of AMP, PZ, and their aqueous blends, including CESAR1, is covered in a recent publication by Morlando et al.^29^ A general overview of previously identified and suggested compounds is given there.

All amines and other organic compounds tend to form the same kind of direct oxidation products, namely, small organic acids, aldehydes, and, specifically for amines, ammonia. Both AMP and PZ are known to form formic, acetic, oxalic, and glycolic acid, and ammonia under oxidizing conditions.^30,31^ These compounds form irreversibly, meaning that the CO_2_ capture capacity of the solvent amine lost through oxidation cannot be regained. These oxidation products are often referred to as heat-stable salts (HSS) and may form secondary degradation products by reacting with other compounds in the solvent, i.e., the solvent amine. HSS are targeted in most solvent reclaiming techniques and can be removed either by thermal reclaiming, ion exchange, or electrodialysis.^7,32^ Removal of HSS does, however, not necessarily reduce the degradation rate of the solvent.^7,33,34^

Volatile degradation compounds are undesirable in the PCC of CO_2_ from industrial sources, as they will require robust emission mitigation technologies to ensure safe operation. The main degradation compound that is usually monitored in the cleaned flue gas is ammonia, which is also formed in CESAR1.^7,12^ During a study of emissions from CESAR1 operation at TCM, 11 additional compounds were monitored in the cleaned flue gas, including formaldehyde, acetaldehyde, and acetone.^11^ Also, the CO_2_ product will either come out contaminated or require further purification if volatile species are present in the solvent, and so far, volatile losses to the CO_2_ stream have not been studied in detail.^35^ As part of the ongoing CO_2_ capture pilot campaigns with CESAR1 in the HEU project AURORA, volatile products coming out of both the absorber and desorber columns are being monitored.

The following sections will briefly cover the current knowledge on degradation of the specific solvent components of CESAR1: AMP and PZ, as well as that of their blend.

### 1.1 AMP Degradation

AMP is among the most stable primary amines under oxidizing conditions that have been tested in the CO_2_ capture context.^6,24^ The steric hindrance around the amino group causes the CO_2_ binding mechanism of aqueous AMP to result in a greater formation of carbonate/bicarbonate than other primary amine systems, making it differ from most other primary amine species.^36,37^ This same steric hindrance is also likely the cause of AMP’s higher oxidative stability compared to other primary amines, as the possibility for chemical reactions to take place around the more rigidly bound amine group is limited.^6^ Contributions to the elucidation of AMP degradation chemistry have been made by several research groups, and many degradation compounds were identified by Wang,^31^ Lepaumier et al.,^23,24^ and Eide-Haugmo et al.^4,5^

In the PhD thesis of Wang,^31^ some of the degradation products of AMP were quantified after thermal and oxidative degradation. The main identified products of thermal AMP degradation were 4,4-dimethyl-2-oxazolidinone (DMOZD), 2-[(2-amino-2-methylpropyl)­amino]-2-methyl-1-propanol (AMPAMP), and 4,4-dimethyl-1-hydroxy-*tert*-butyl-2-imidazolidinone (DM-HTBI). After oxidative AMP degradation, the main products were suggested to be ammonia, 2,4-lutidine (LUT), DMOZD, and formic acid (FAc). The sum of quantified nitrogen-containing oxidation products (DMOZD, NH_3_, LUT, nitrite, and nitrate) in that work accounted for approximately 57% of the N lost from AMP to degradation.^31^ Wang and Jens^38^ also proposed further oxidative and thermal AMP degradation products. Analysis for LUT in this work concluded that LUT is not present in significant amounts during either AMP or CESAR1 degradation.

### 1.2 PZ Degradation

PZ has been extensively studied both alone and in blends for use in PCC applications. Many studies have examined various aspects affecting its degradation, i.e., effect of temperature, loading, oxygen content, and metal content, and also degradation mitigation technologies have been studied in aqueous PZ.^30,39–41^

Freeman^30^ found FAc, *N*-formyl piperazine (FPZ), and ethylenediamine (EDA) to form during PZ oxidation and proposed many further compounds as likely PZ degradation products. During oxidative PZ degradation, Wang^31,42^ found NH_3_, EDA, piperazinone (OPZ), FPZ, FAc, oxalic acid, and oxalyl amides as the main degradation products. The sum of quantified nitrogen-containing oxidation products (EDA, FPZ, OPZ, *N*-nitrosopiperazine (MNPZ), NH_3_) in this work accounted for approximately 47% of the N lost from PZ due to degradation.^42^

PZ has been found to be quite stable under thermal stress, but, as for other amines, the degradation increases with temperature, loading, and PZ concentration. The degradation reaction is proposed to be initiated through a nucleophilic attack, resulting in ring opening of PZ.^30^ Although not formed to any large extent at the pilot scale, some of the main thermal degradation products have been identified as FPZ, ammonia, *N*-(2-aminoethyl)-piperazine (AEP), EDA, *N*-(ethyl)-piperazine (EPZ), 2-imidazolidone, and FAc.^43–45^

### 1.3 AMP/PZ Blend Degradation

In addition to producing the degradation compounds found after degradation of the individual solvent amines, CESAR1 will also produce compounds that form as a result of both solvent amines being present and reacting with one another. The literature so far has suggested some compounds that may form in the CESAR1 blend, but none had been positively identified or quantified in the degraded solvent before.^31,46^ Further, beyond the possibility of forming new blend degradation products, blends of amines also tend to degrade faster than the sum of their individual components.^47^

Pilot campaigns with CESAR1 that have studied the formation of degradation products have found that significant concentrations of both AMP- and PZ-derived degradation compounds are present in the solvent, and additionally, propionic acid (PAc) has been seen to form during CESAR1 degradation.^7,8^ Notably, the CESAR1 solvent is much more stable than 30 wt % MEA (*aq.*) under PCC conditions.^7,8^ Additionally, CESAR1 exhibits a much more predictable and linear degradation profile, easing operation and reducing the likelihood of unexpected degradation incidents that could lead to unplanned downtime and loss of capacity.^7,8,33,48^

### 1.4 Nitrosamine and Nitramine Formation

Formation of nitrosamines and nitramines is one of the largest concerns in terms of emissions from CO_2_ capture plants due to their highly carcinogenic nature.^49,50^ As nitrosamines have been identified as potentially harmful compounds that may form during the CO_2_ capture process, large research efforts have been dedicated to assessing their impact on health and their fate in the environment. Because of this, these groups of compounds already have strict emission limits, something which is not true for all degradation compounds formed in the process.^51–57^ The emission limits necessitate monitoring these species in the emitted flue gas and in the local environment, such as drinking water sources. Monitoring these and other harmful compounds is important to ensure the safety of plant operators and others who may work with or come into contact with the solvent, water, or acid wash solutions from the process.

Nitrosamines and nitramines have the general structures —N—N=O and −N–NO_2_, respectively. During plant operation, stable nitrosamines can form from secondary amines reacting with residual NO_*X*_ in the flue gas. Although this means that solvents containing secondary amines are especially susceptible to nitrosamine formation, other amines can also indirectly form nitrosamines. This happens through the formation of secondary amine degradation products, which then, in turn, can undergo nitrosation and form nitrosamines. Formation of nitrosamines has been reported for PZ and CESAR1.^58^ As PZ is a secondary amine, both PZ and CESAR1 readily form stable nitrosamines when exposed to aqueous NO_2_. Nitrosamines formed during PZ nitrosation are mainly MNPZ, with smaller amounts of *N*,*N*’-dinitrosopiperazine (DNPZ). AMP forms nitrosamines to a much smaller extent than PZ, since AMP is a primary amine. However, this work shows that *N*-methyl AMP (MAMP), which is usually present in AMP in small amounts, readily forms stable *N*-nitroso methyl AMP (NMAMP). Nitramines can form through any amine coming into contact with NO_2_. Nitramines are generally significantly less carcinogenic than the corresponding nitrosamines and are typically found in lower concentrations.^59^

In this work, we present the degradation chemistry of AMP, PZ, and CESAR1 at temperatures and CO_2_ loadings relevant to the absorption and desorption steps of the postcombustion CO_2_ capture process to elucidate the formation paths of various degradation compounds found in samples from pilot plant operations.^28^

## 2 Materials and Methods

### 2.1 Chemicals

2-Amino-2-methyl-1-propanol (AMP, CAS: 124–68–5, Acros Organics) and piperazine (PZ, CAS: 110–85–0, Sigma-Aldrich), both with 99% purity, were used to prepare the solvents in this work. Additionally, Sep-Pak DNPH-Silica Cartridges from Waters were used to capture ketones and aldehydes. Each cartridge contains 800 mg of 2,4-dinitrophenylhydrazine (DNPH, CAS: 119–26–6) in amorphous silica (CAS: 63231–67–4) and acetonitrile (CAS: 75–05–8).

For the preloading of solvents and makeup CO_2_ during oxidative degradation, CO_2_ (IND.) from AGA/Linde was used. Air for the oxidative degradation experiment was compressed using an in-house compression system at SINTEF. CO_2_ loading, α, is defined as moles of CO_2_ per mole of amine functionality (= N in the fresh solvent), as given in eq 1. One mole of AMP, therefore, counts as one mole of amine, and one mole of PZ counts as two.

1 $$\alpha =\frac{n_{{CO}_{2}}}{n_{alkalinity}}$$

All solvents used in this work were prepared gravimetrically, and the actual concentrations were subsequently verified by TIC analysis and titration.

### 2.2 Oxidative Degradation Experiments

Oxidative degradation of the single amines and CESAR1 blend was performed according to Vevelstad et al.^60^ in a water bath-heated double-jacketed glass reactor at 60 °C, with 357.5 mL/min gas (77% N_2_, 21% O_2_, 2% CO_2_ dry) sparged into 1 L of solvent, gas phase recycle of 50 L/min and constant magnetic stirring to eliminate mass transfer limitations. The solvent was preloaded with CO_2_ at the beginning of the experiment to contain 0.4 mol CO_2_ per mol amine functionality (mol_CO2_/mol_N_), and iron sulfate (FeSO_4_, 0.5 mmol/L) was added to catalyze the oxidation reactions. The added dry gas blend is led through a cooled water saturation chamber prior to being sparged through the solvent, while the bleed leaving the reactor flows through two 40 cm double-jacketed Graham condensers before exiting through a series of two impinger bottles containing 0.1 M H_2_SO_4_ (aq.). Samples were taken from the solvent two times per week. A DNPH cartridge was attached between the second condenser and the first impinger bottle for the last 4 days of the experiment. Solvent samples, acid wash solutions, and DNPH cartridges were analyzed after the completion of each experiment. An overview of the oxidative degradation experiments is shown in Table 1. The prepared solvent solutions were also analyzed for degradation compounds prior to stress testing to quantify any presence of degradation compounds in the fresh solvent samples.

**Table 1:** Conditions Used for Various Degradation Experiments of the Different Amine Solutions

	AMP	PZ		α (mol_CO2_/mol_N_)			Temperature (°C)			
System	(mol/kg)			0.1	0.4	0.6	60	120	135	150
Oxidative degradation										
AMP	3.0		-	-	X	-	X	-	-	-
PZ	-		1.5	-	X	-	X	-	-	-
CESAR1	3.0		1.5	-	X	-	X	-	-	-
Thermal degradation										
AMP	3.0		-	-	X	-	-	X	X	X
PZ	-		1.5	-	X	-	-	X	X	X
CESAR1	3.0		1.5	X	X	X	-	X	X	X

### 2.3 Thermal Degradation Experiments

Testing of the thermal stability of AMP, PZ, and CESAR1 was performed in closed SS316L cylinders according to Lepaumier et al.^61^ over a period of 28 days. For the CESAR1 solvent, one sample was taken after 10 days and another after 19 days by removing one cylinder from the convection oven. For all solvents, two cylinders were removed after 28 days, with the exception of CESAR1 with α = 0.6 mol_CO2_/mol_N_ at 150 °C, where only one cylinder could be used. The variance of the compounds quantified in the two parallels for each experimental condition was used as the uncertainty for the results of each experiment. CESAR1 was tested at three different temperatures and CO_2_ loadings, and 3.0 mol/kg AMP and 1.5 mol/kg PZ with 0.4 mol of CO_2_ per mol of nitrogen were tested at 3 different temperatures, as described in Table 1.

### 2.4 Analytical Methods

#### 2.4.1 CO_2_ and Nitrogen Quantification

Analysis of total CO_2_ and total nitrogen (TN) concentrations in the samples was performed on a Shimadzu TOC-L_CPH_ with a TNM-L detector. For the CO_2_ quantification, the instrument was used in total inorganic carbon (TIC) mode, where the diluted amine sample is injected into a vessel of phosphoric acid (H_3_PO_4_, 25 wt %, *aq.*) that is sparged with air, releasing all inorganic carbon as CO_2_. The CO_2_ is quantified using its response from a nondispersive infrared (NDIR) detector and calibrated with sodium bicarbonate (NaHCO_3_). For the quantification of total nitrogen, the diluted sample is led into a Pt catalyst at 720 °C, turning all nitrogen into nitric oxide (NO), which is reacted with ozone (O_3_) to form excited nitrogen dioxide (NO_2_*), which is quantified using a chemiluminescence detector and calibrated with standards made from pure (>99%) MEA. The total alkalinity of each sample was measured by titration with sulfuric acid (H_2_SO_4_, 0.1 mol/L, *aq.*) according to Ma’mun et al.^62^

#### 2.4.2 Liquid Chromatography with Mass Spectrometry (LC-MS)

Nearly 100 degradation compounds, either proposed in the literature or suggested based on analogous degradation mechanisms known for amines other than the CESAR1 components, were evaluated. Some of these compounds were already available in the analytical method park at SINTEF, while standards for some selected compounds were purchased and methods were developed for their analysis in the first attempt to close the nitrogen balance of degraded CESAR1 samples. Compounds were selected first based on the literature review by Morlando et al.,^29^ their likelihood of formation during degradation, and second based on the possibility of obtaining analytical standards for method development. Analytical standards were partly purchased from known chemical vendors, i.e., Merck Life Science, and partly synthesized on demand by Chemsupport AS. The compounds (more than 50) for which methods were developed and/or were available for quantitative LC-MS/MS analysis are given in Tables 2 and 3. While Table 2 lists compounds found in the degraded CESAR1, AMP, and PZ samples, Table 3 lists the hypothesized degradation compounds for AMP, PZ, or CESAR1 that were below the limit of detection or quantification (LOD/LOQ) in all samples. Some of these compounds were specifically selected based on suggestions in the literature, such as LUT, DAEP and HMTA, while others, i.e., the organic acids, were already included in some of the LC-MS/MS methodologies used to study other compounds.

**Table 2:**
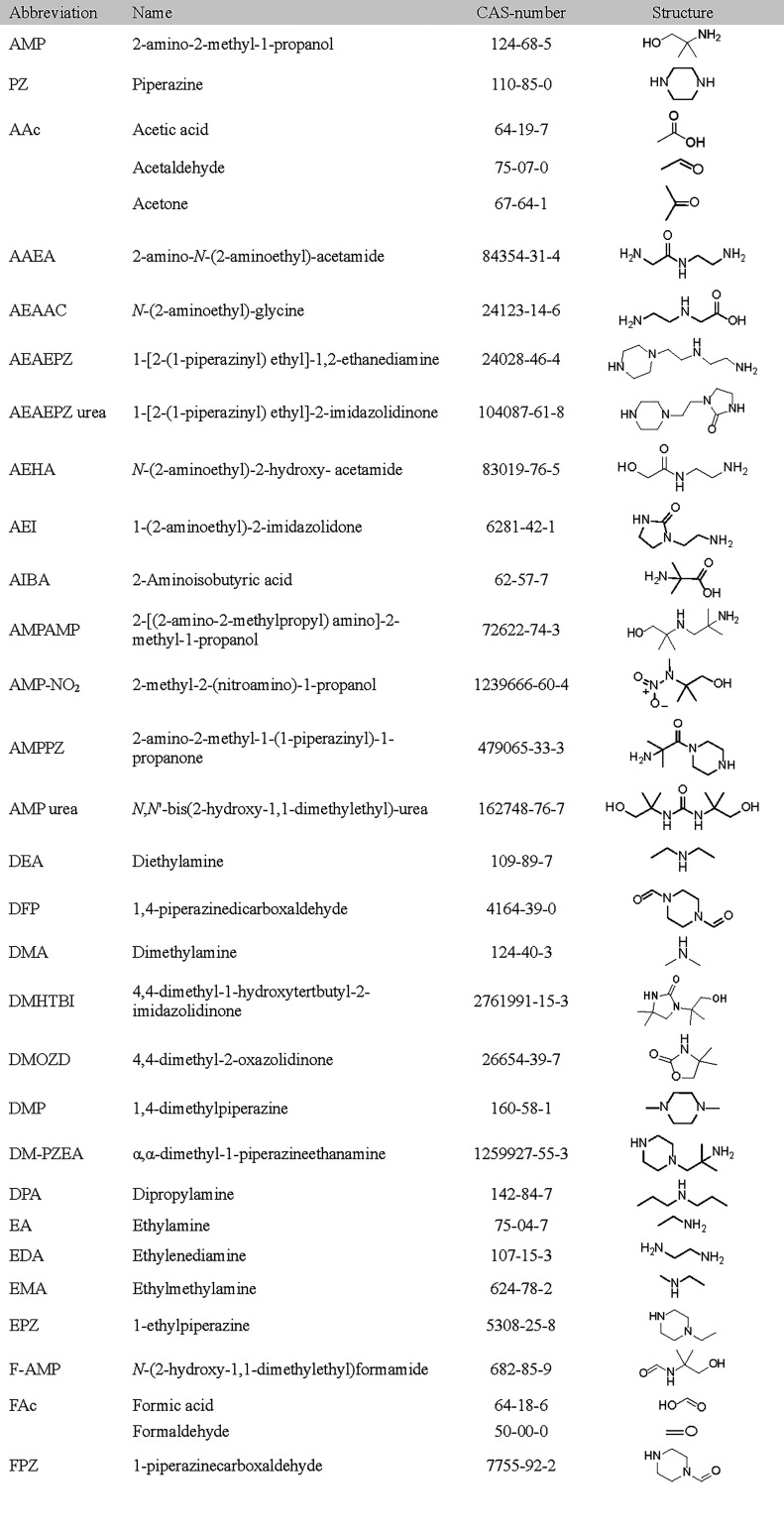

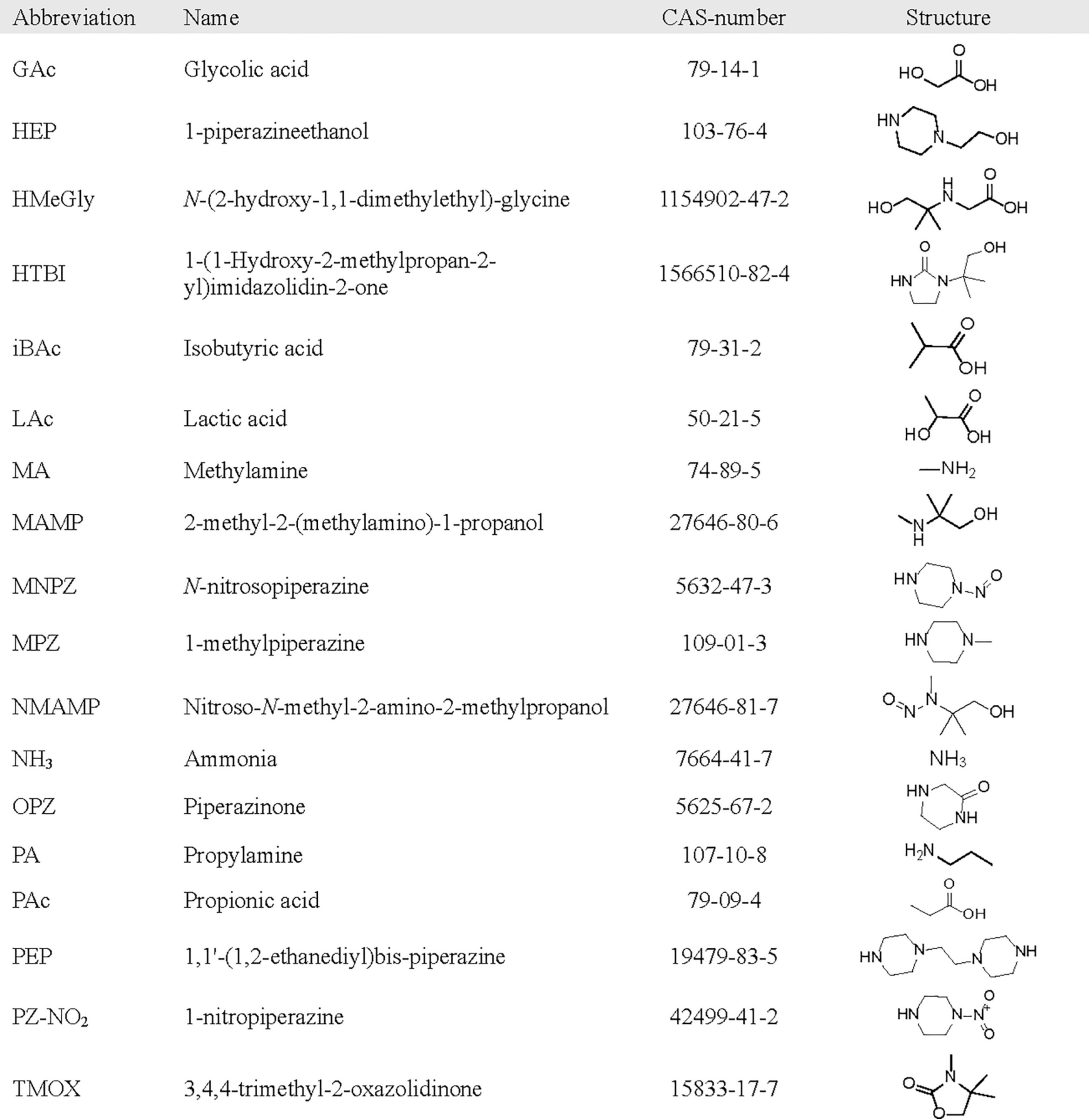
Compounds Found in Degraded CESAR1, AMP, and PZ Solvent Samples Using Various LC-MS Techniques

**Table 3:**
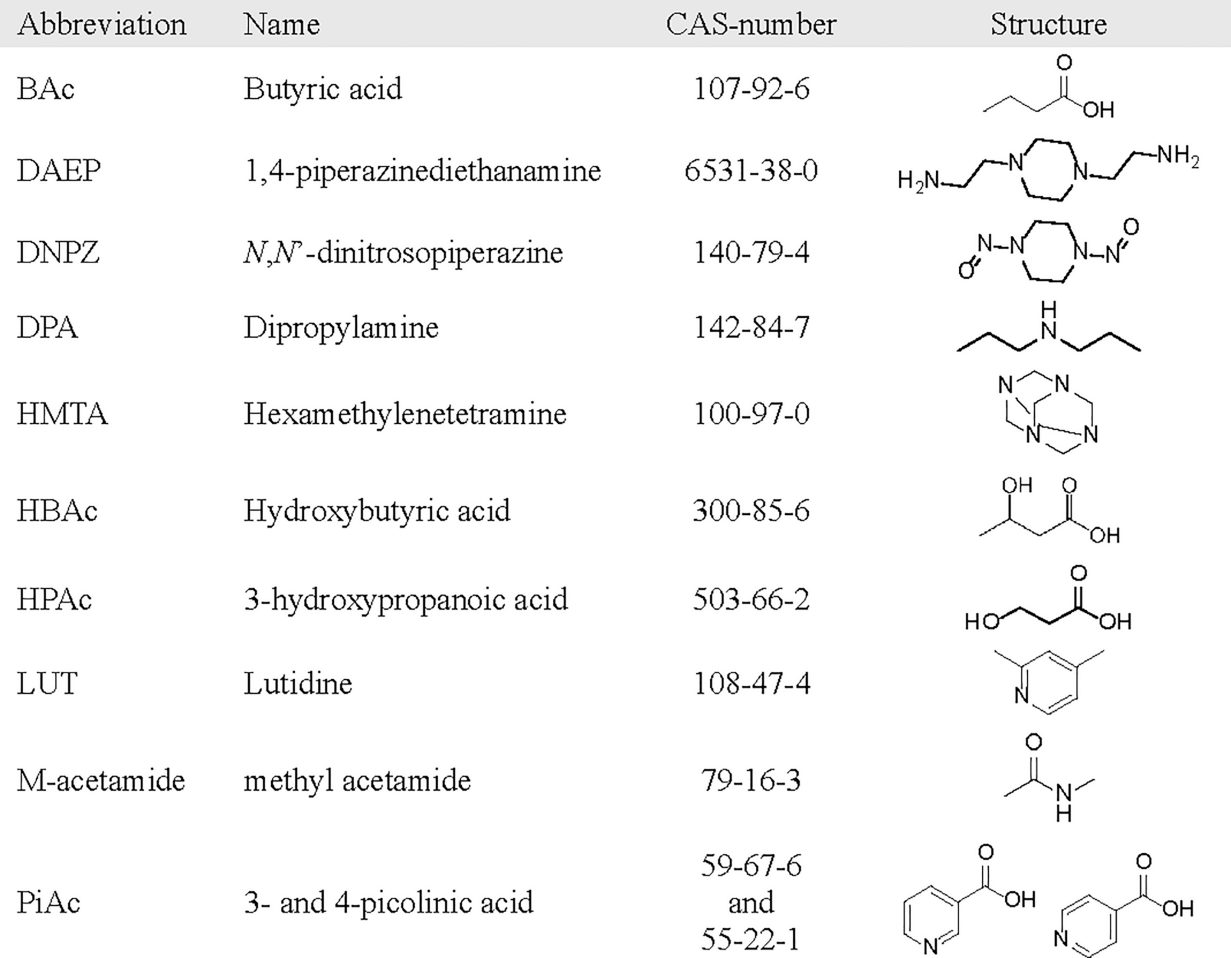
Compounds That Were Studied in This Work but Not Found above the LOQ of the LC-MS/MS Methods in Any of the Studied Samples

## 3 Results and Discussion

As listed in Tables 2 and 3, more than 50 degradation compounds were analyzed in all samples. Even though all compounds seen in the single amine laboratory experiments are also present in the CESAR1 blend, there are also degradation components that are seen only for the blend. This clearly shows that the degradation is not only a sum of the degradation of each component.

It was found that the fresh solvent samples contained some degradation products or trace contaminants even before undergoing thermal or oxidative degradation. This could either be due to minor degradation taking place during storage, or they could be present as byproducts of the synthesis of AMP and PZ. AMP and CESAR1 contained MAMP (7–15 mmol/kg), F-AMP (0.03–0.2 mmol/kg), AMPAMP (0.01–0.04 mmol/kg), methylamine (0.05 mmol/kg), and ammonia (0.8–1.1 mmol/kg). PZ and CESAR1 contained OPZ (0.3–0.6 mmol/kg) and formaldehyde (0.1–5 mmol/kg).

### 3.1 Thermal Degradation of CESAR1

Table 4 shows the average loss of the two solvent amines with different solvent compositions at different temperatures after 4 weeks of thermal stress. PZ is observed to be slightly more stable under thermal stress than AMP, especially when used alone. This observation agrees with the scientific literature.^4,63^ In the CESAR1 blend, the stability of PZ is slightly higher compared to AMP, except at a loading of 0.1 mol_CO2_/mol_N_, meaning PZ losses are slightly lower than those of AMP, but the losses are generally in the same order of magnitude for both amines in CESAR1. Thermal AMP degradation seems to be slightly more dependent on the CO_2_ loading than thermal PZ degradation, but both amines display increased degradation with increasing CO_2_ loading/concentration.

**Table 4:** Loss of AMP and PZ after 28 Days of Thermal Degradation of the Different Solvent Compositions at Different Temperatures

	120 °C		135 °C		150 °C	
	AMP 3.0 M	PZ 1.5 M	AMP 3.0 M	PZ 1.5 M	AMP 3.0 M	PZ 1.5 M
AMP 3.0 M α = 0.4	7%		15%		27%	
PZ 1.5 M α = 0.4		4%		4%		5%
CESAR1 α = 0.1	2%	5%	5%	6%	16%	20%
CESAR1 α = 0.4	6%	6%	20%	18%	48%	46%
CESAR1 α = 0.6	13%	7%	28%	23%	50%	43%

All of the AMP and CESAR1 solvents contained 7–15 mmol/kg of MAMP before they were subjected to thermal stress. From Figure 1a–c, it is evident that MAMP is not very stable under the given conditions and exhibits losses between 86% (AMP, 120 °C) and 97% (CESAR1, 150 °C) after 28 days. MAMP degradation rates increase with an increase in temperature and CO_2_ loadings. In the thermal degradation experiment with CESAR1 at 150 °C and α = 0.1 mol_CO2_/mol_N_, only 3% of the MAMP present in the initial sample remained at the end of the experiment. None of the test conditions in this work gave increasing MAMP concentration, indicating that its presence is not due to solvent degradation.

**Figure 1:**
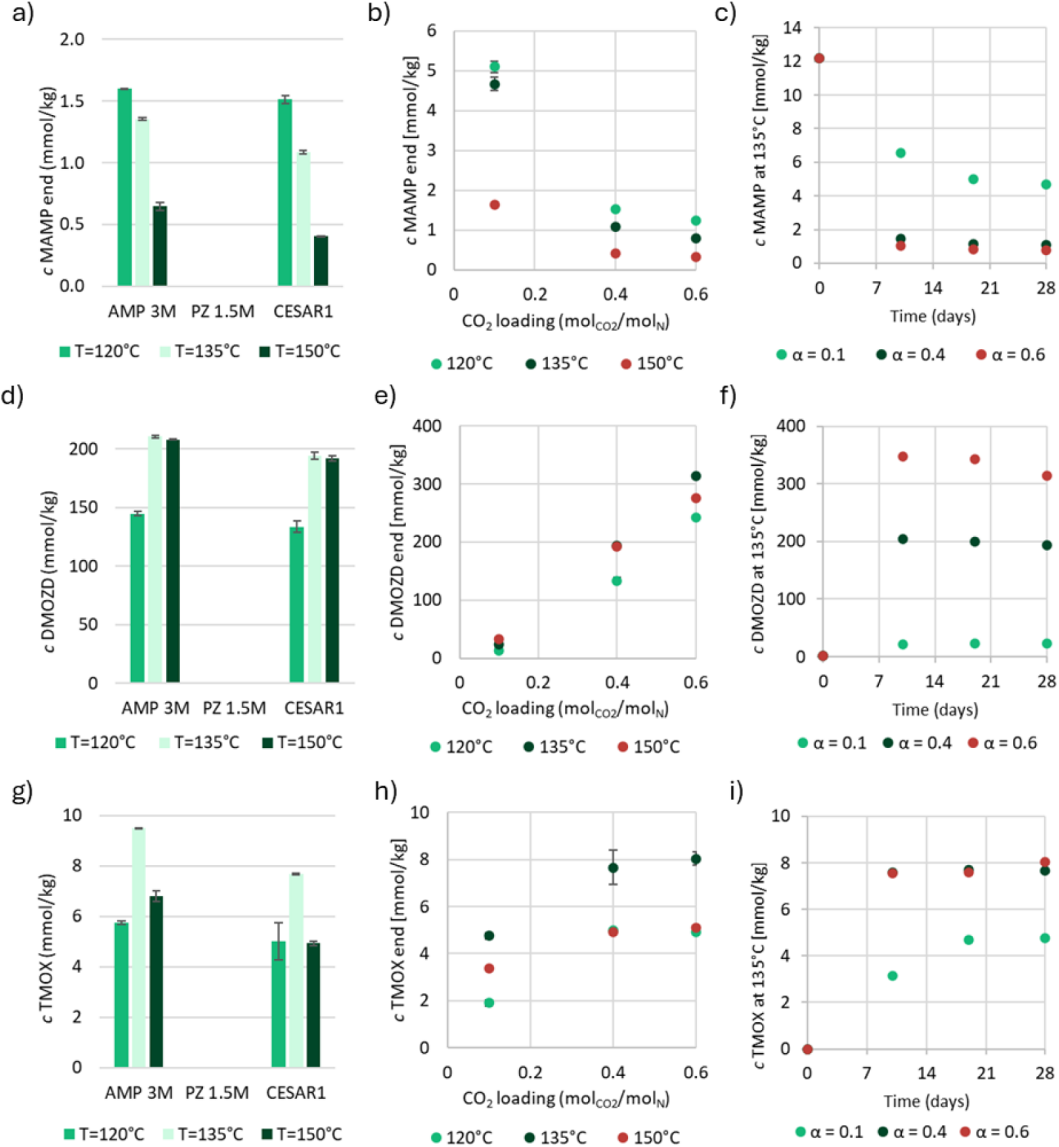
a) MAMP, d) DMOZD, and g) TMOX concentrations in thermally degraded AMP, PZ, and CESAR1 solvents (α = 0.4 mol_CO2_/mol_N_) after 28 days at three different temperatures. b) MAMP, e) DMOZD, and h) TMOX concentrations in the thermal degradation experiments with CESAR1 after 28 days, as a function of CO_2_ loading, and c) MAMP, f) DMOZD, and i) TMOX as a function of time at 135 °C.

In Figure 2, an overview of all of the degradation products that were found and quantified in thermally degraded CESAR1 with a CO_2_ loading of 0.4 mol_CO2_/mol_N_ is shown. The two most abundant products, DMOZD and DM-PZEA, are present in concentrations at least 1 order of magnitude higher than the remaining compounds. Regardless of temperature, these two are the main thermal degradation products of CESAR1 under the given conditions. Of the 37 compounds found above the LOQ in thermally degraded CESAR1, seven compounds have not previously been quantified in PZ, AMP, or any blend of the two in the open literature. These compounds are DM-PZEA, TMOX, AEAEPZ, AEAEPZ-urea, AMP-urea, HTBI, and PEP. Additionally, the two organic acids LAc and iBAc were found in the thermally degraded samples, which seem to require a combination of oxidizing conditions and high temperature to form. The following section will discuss the different compounds, how they are most likely formed, and how CO_2_ loading and temperature impact their formation.

**Figure 2:**
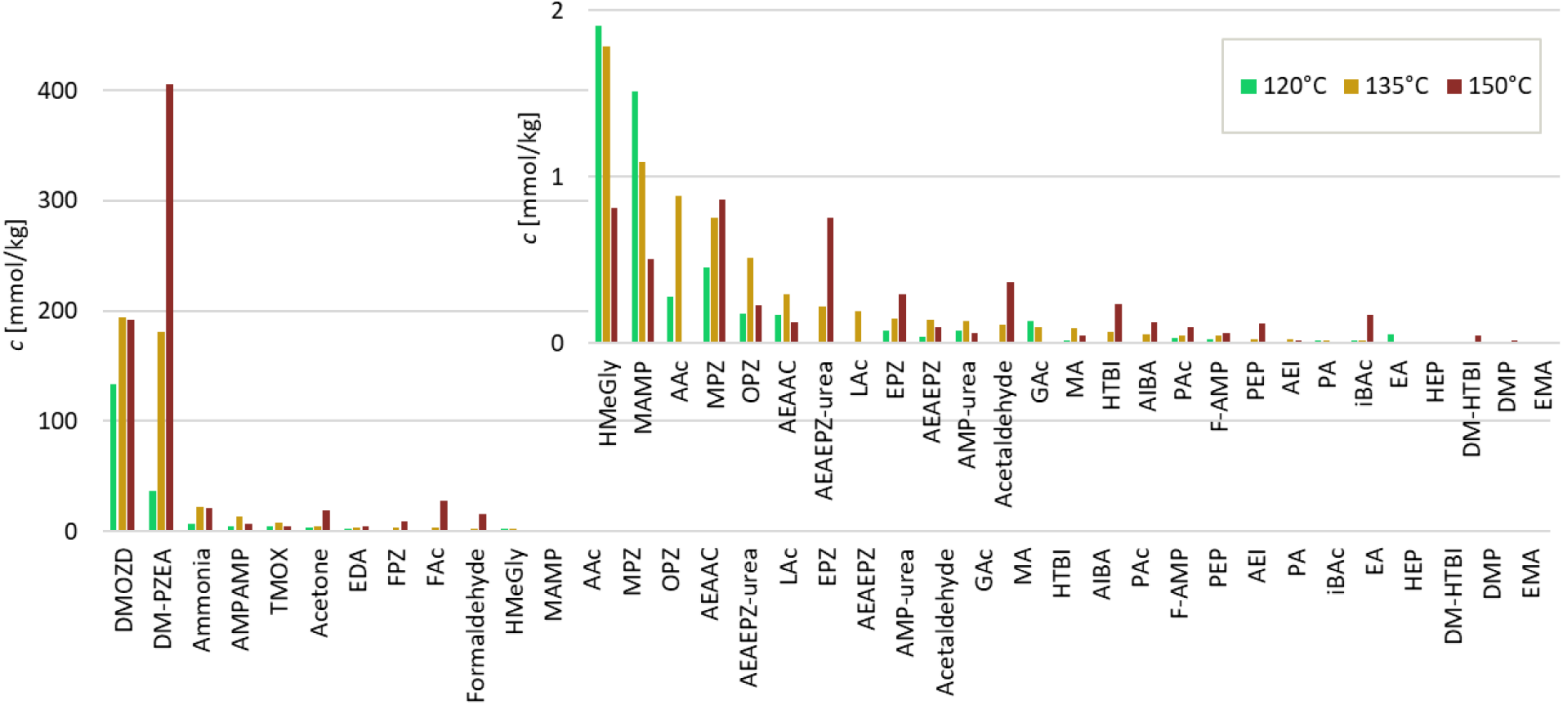
All thermal degradation products (and MAMP) quantified in CESAR1 with α = 0.4 mol_CO2_/mol_N_ at three different temperatures for 28 days.

As is the consensus in earlier literature,^23,31,38^ our study also shows that DMOZD is a dominant thermal degradation product of AMP. Its formation is expected to happen through cyclization of the AMP carbamate, as depicted in Scheme 1. DMOZD is also one of the most abundant thermal degradation compounds found in CESAR1.

**Scheme 1:**
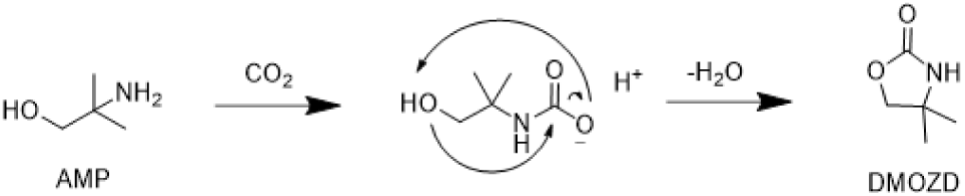
AMP Carbamate Likely Forms DMOZD through a Cyclic Condensation Reaction According to Lepaumier et al.^23^

Figure 1d shows that the concentration of DMOZD is the same in AMP and CESAR1 after thermal degradation, and that DMOZD does indeed not form in PZ. DMOZD formation is highly dependent on the CO_2_ loading, as illustrated in the two graphs in Figure 1e–f. Figure 1f shows that a steady-state or maximum concentration of DMOZD is reached rapidly in all experiments, and no concentration change is observed after the first sample taken on day 10, indicating that DMOZD either further reacts or is in equilibrium with other species. Up to 9% of AMP is converted to DMOZD during thermal degradation at 120 °C, while less than 0.1% reacts to form DMOZD during oxidative degradation of AMP, making DMOZD a thermal degradation product of AMP, as previously reported in the literature.^5,23,31^ At 120 and 135 °C, DMOZD is the most dominant thermal degradation product of both AMP and CESAR1.

As already mentioned, MAMP, which is present in fresh AMP, and CESAR1 also undergo thermal degradation. The AMP used in the laboratory-scale degradation tests in this work contained approximately 0.25 mol % MAMP relative to AMP (*c*_MAMP_/*c*_AMP_). Its primary degradation route under thermal degradation is the formation of TMOX, as illustrated in Scheme 2. This condensation reaction is analogous to DMOZD formation from AMP.

**Scheme 2:**
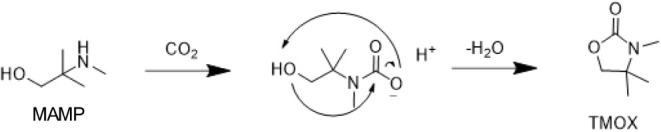
Suggested Mechanism of the Formation of TMOX from MAMP

TMOX concentrations during thermal degradation of AMP alone and in the CESAR1 blend are very similar, as can be seen in Figure 1g. Figure 1h depicts the TMOX concentration as a function of CO_2_ loading and clearly illustrates that the highest TMOX concentration is observed at 135 °C, regardless of the CO_2_ concentration. TMOX formation also depends on the CO_2_ loading, as shown in Figure 1i, where loadings higher than α = 0.1 mol_CO2_/mol_N_ increase TMOX concentrations in CESAR1. The two highest loadings show no significant difference in their TMOX concentrations, and the formation seems to plateau during thermal degradation (at 135 °C), as depicted in Figure 1i. It is likely that both DMOZD and TMOX react to form further degradation compounds through secondary reactions at 150 °C, which is why the concentration after this experiment appears to be very similar to that at 135 °C and not higher. During thermal degradation at 120 °C, up to 80% of the MAMP has been converted into TMOX, while at 150 °C, less than 1% of the initial MAMP is left in the samples with the highest loading.

The likely pathway for the formation of the thermal degradation product DM-PZEA is based on a nucleophilic attack by PZ on DMOZD, as presented in Scheme 3. This is in line with the suggested formation of an analogue compound in an MEA/PZ blend^31,64^ and the general carbamate polymerization reaction seen in CO_2_-loaded amine solvents during thermal degradation.^23,65^

**Scheme 3:**
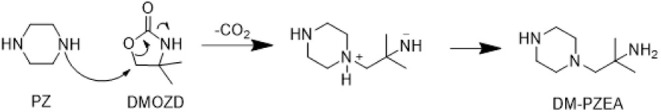
Suggested Mechanism for the Reaction of PZ with DMOZD at High Temperature to Form the Polymerization Product DM-PZEA

Figure 3a confirms that DM-PZEA does not form in AMP or PZ alone but requires the presence of both amines. A higher temperature seems favorable for DM-PZEA formation, as depicted in Figure 3a–b, where each temperature increase gives a higher concentration of DM-PZEA. Figure 3b–c shows that a higher CO_2_ loading is also favorable for DM-PZEA formation. This supports the proposed mechanism in Scheme 3 as it requires the presence of CO_2_ in the form of DMOZD. The rate of formation or accumulation of DM-PZEA does not significantly differ between CO_2_ loadings of 0.4 and 0.6, but more PZ-DMEA is found in these experiments than in those with α = 0.1 mol_CO2_/mol_N_.

**Figure 3:**
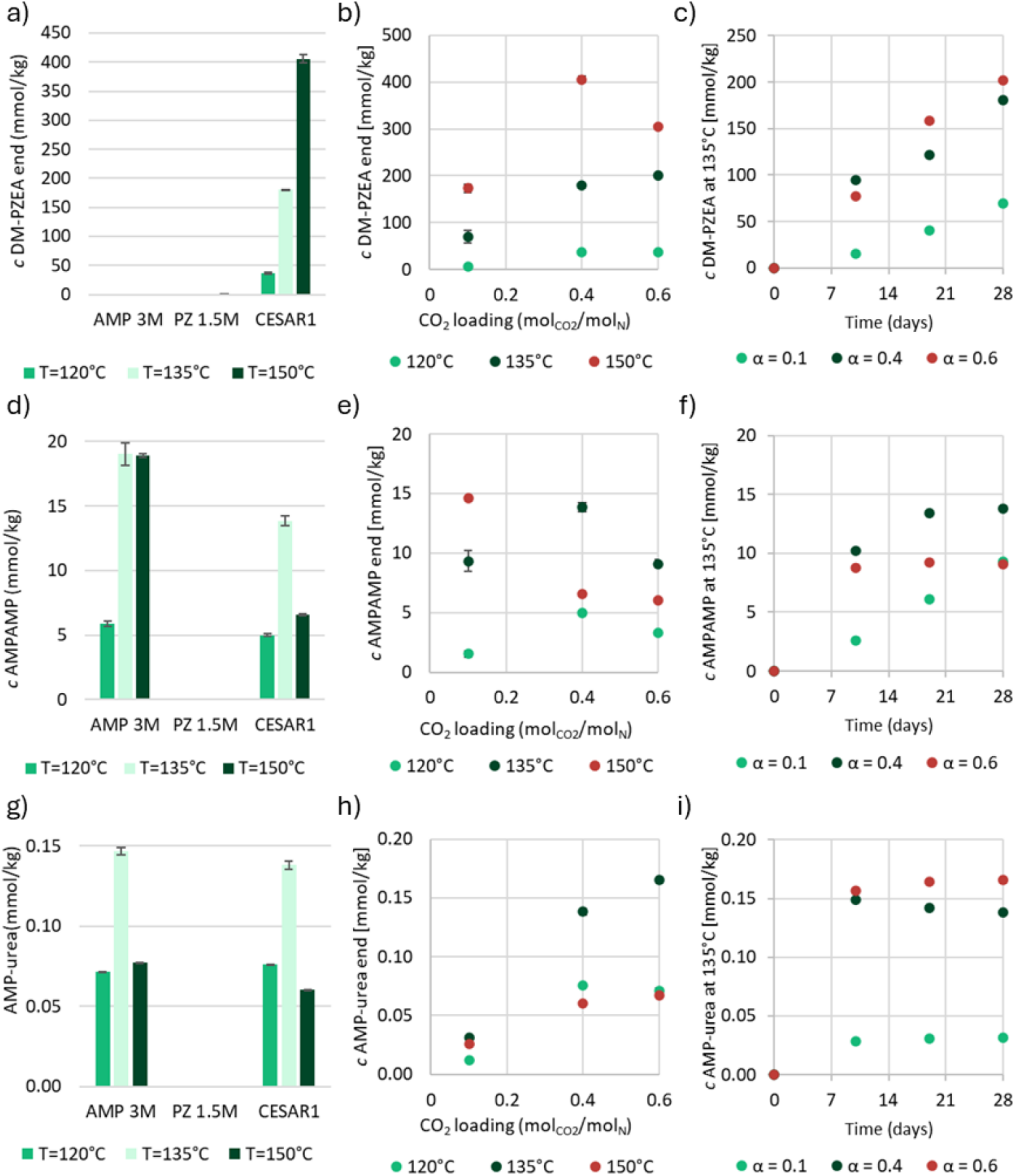
a) DM-PZEA, d) AMPAMP, and g) AMP-urea concentrations in thermally degraded AMP, PZ, and CESAR1 solvents (α = 0.4 mol_CO2_/mol_N_) after 28 days at three different temperatures. b) DM-PZEA, e) AMPAMP, and h) AMP-urea concentrations in the thermal degradation experiments with CESAR1 after 28 days, as a function of CO_2_ loading, and c) DM-PZEA, f) AMPAMP, and i) AMP-urea as a function of time at 135 °C.

AMPAMP is a dimer of AMP, likely formed through the carbamate polymerization mechanism with a ring opening of DMOZD, similar to the formation of DM-PZEA, as presented in Scheme 4. AMPAMP is formed to a much lesser extent than DM-PZEA, as AMP is less prone to undergo the nucleophilic attack due to its steric hindrance.

**Scheme 4:**
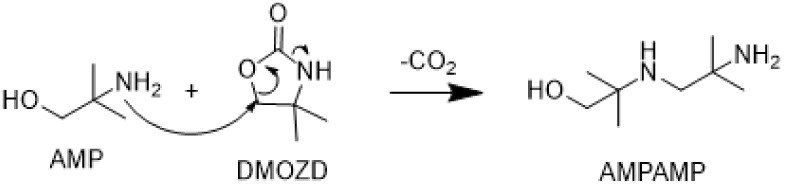
Analogue to the Formation of DM-PZEA, AMP is Suspected to Act as the Nucleophile Attacking DMOZD to Form AMPAMP

Slightly more AMPAMP is found in AMP compared to CESAR1 above 120 °C after thermal degradation, as shown in Figure 3d. This is likely caused by fewer competing reactions involving DMOZD in the pure AMP solvent. Figure 3e–f shows that there is no simple correlation between the CO_2_ loading or temperature and AMPAMP concentration in the CESAR1 solvent. At the lowest loading, the highest temperature results in the highest AMPAMP concentration. At higher loadings, however, the AMPAMP concentration is the highest at 135 °C. This indicates that AMPAMP is an intermediate reacting to form further degradation products, especially at higher temperatures. One possibility is the formation of longer polymers through a similar ring-opening reaction or formation of DM-HTBI through intramolecular carbamate condensation.

Ureas of amines are likely to form through a polymerization/condensation reaction between an oxazolidinone (i.e., DMOZD) and another amine, as demonstrated by Yazvikova et al. in MEA.^66^ AMP-urea forms during thermal degradation of both AMP and CESAR1, likely according to Scheme 5.

**Scheme 5:**
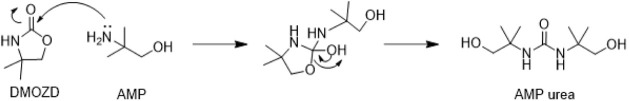
Formation Mechanism of the Urea of AMP

Figure 3g shows that the tendency to form AMP-urea is the same in pure AMP and the CESAR1 solvent, and that the highest concentration of AMP-urea is found at 135 °C. It is likely that AMP-urea itself is unstable at 150 °C and further reacts or decomposes, which is why the concentration of AMP-urea is lower at this temperature than at 135 °C. Figure 3h confirms that this happens over the whole CO_2_-loading range. Figure 3i shows that the AMP-urea concentration plateaus early, which indicates that AMP-urea might exist in equilibrium with other species, whether through reacting further or through the reverse formation reaction. There is a slight dependency on the CO_2_ loading, but no significant difference is found between α = 0.4 and 0.6 mol_CO2_/mol_N_. This was also the case at 120 and 150 °C (data are provided in the Supporting Information).

AEAEPZ and AEAEPZ-urea are formed as a result of thermal degradation of PZ. AEAEPZ forms to a larger extent in PZ compared to CESAR1, as shown in Figure 4a, while Figure 4d shows that AEAEPZ-urea is present in similar concentrations in both PZ and CESAR1. In Figure 4b–c, it can be seen that a lower CO_2_ loading seems favorable for the formation of AEAEPZ-urea.

**Figure 4:**
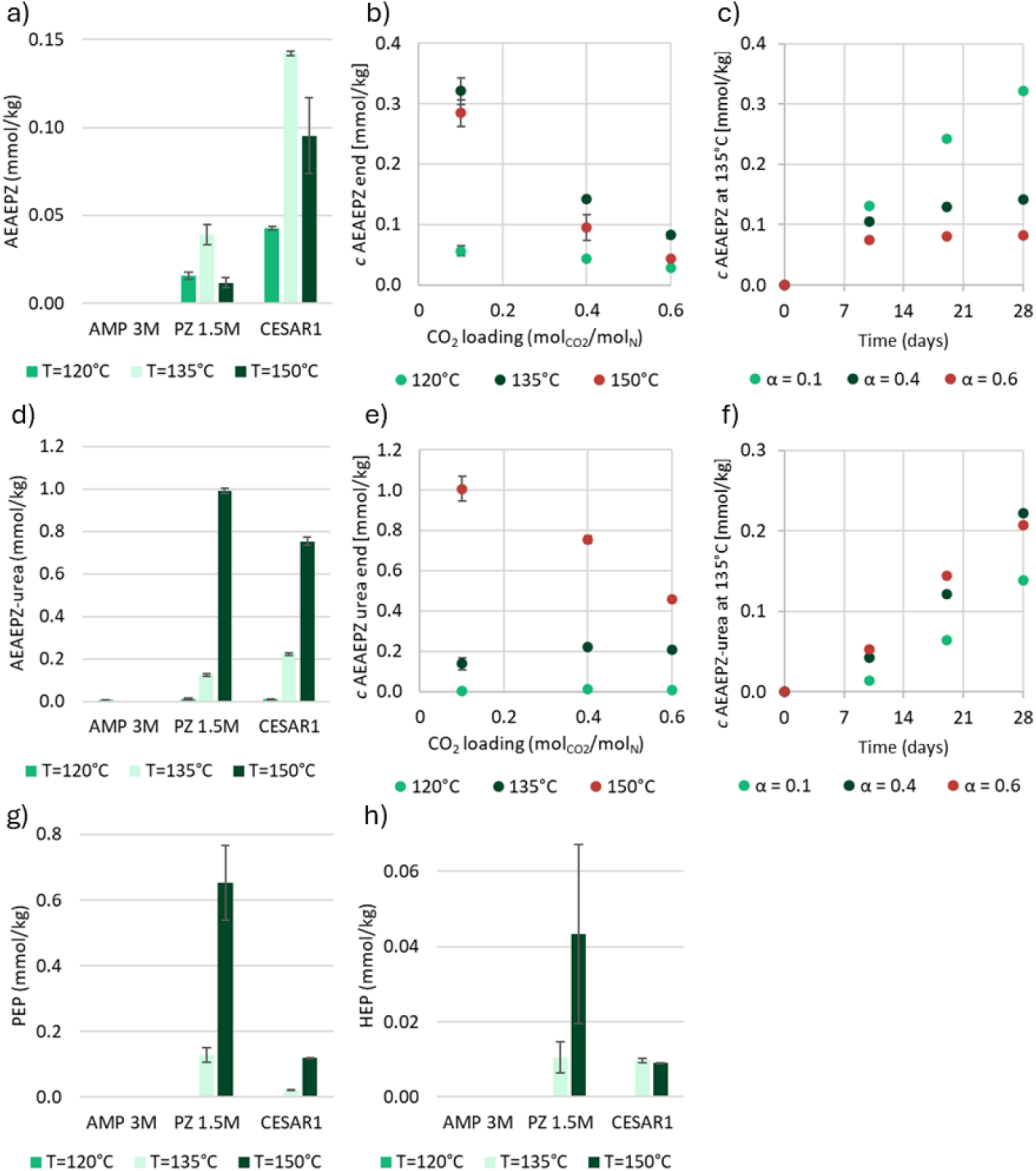
a) AEAEPZ, d) AEAEPZ-urea, g) PEP, and h) HEP in thermally degraded AMP, PZ, and CESAR1 solvents (α = 0.4 mol_CO2_/mol_N_) after 28 days at three different temperatures. b) AEAEPZ and e) AEAEPZ-urea concentrations in the thermal degradation experiments with CESAR1 after 28 days, as a function of CO_2_ loading, and c) AEAEPZ and f) AEAEPZ-urea as a function of time at 135 °C.

The mechanism proposed in Scheme 6, where AEAEPZ is formed without the involvement of any CO_2_ through PZ carbamate, is supported by the fact that AEAEPZ formation is favored at lower CO_2_ loading. An increase in loading will decrease the reaction rate of this mechanism because more PZ would be occupied as PZ carbamate, leaving less free PZ to react. Figure 4c shows that AEAEPZ accumulation takes place steadily over 28 days at 135 °C, particularly in the CESAR1 solution with a low CO_2_ loading. The same trend is clear at the other two studied temperatures.

**Scheme 6:**
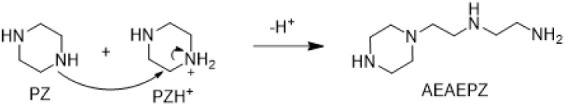
Reaction Mechanism for the Formation of AEAEPZ, as Proposed by Freeman and Rochelle.^43^

Freeman and Rochelle^43^ proposed that AEAEPZ can form AEAEPZ-urea from AEAEPZ carbamate, as shown in Scheme 7. AEAEPZ-urea is found in concentrations of about 1 order of magnitude higher than AEAEPZ.

**Scheme 7:**
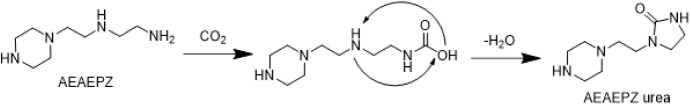
Reaction Mechanism for the Formation of AEAEPZ-Urea, as Proposed by Freeman and Rochelle^43^

AEAEPZ-urea formation is favored at higher temperatures, with much higher concentrations observed in the CESAR1 solvent run at the highest temperature than at the lower temperature. This explains why AEAEPZ is less abundant at 150 °C, as it likely reacts to form more urea. AEAEPZ should be quite reactive, especially in forming the carbamate, as it is a nonsterically hindered primary amine. Because of this, the formation rates could be expected to increase with increasing concentration of CO_2_. The formation of the precursor, AEAEPZ, is, however, hindered by excess CO_2_ which, in turn, would lower the overall formation of AEAEPZ-urea at high loadings. As seen in Figure 4d–f, the combination of a low CO_2_ loading (α = 0.1 mol_CO2_/mol_N_) and a high temperature (150 °C) gives the highest concentration of AEAEPZ-urea. At the lower temperatures, the loading does not have much effect.

PEP and HEP, which are found in very low concentrations in thermally degraded PZ and CESAR1, are also suggested to be formed via AEAEPZ by Freeman and Rochelle.^43^ Both are more abundant in the aqueous PZ solvent alone than in CESAR1, as can be seen in Figure 4g–h. Both PEP and HEP formations depend on CO_2_ loading, where PEP is more abundant at lower CO_2_ loadings, while HEP is more abundant at higher CO_2_ loadings (data are available in the Supporting Information).

MPZ and EPZ are formed during thermal degradation of PZ. As illustrated in Figure 5a–d, a slightly larger amount of MPZ is formed in CESAR1 compared to that in PZ alone under the same conditions, while EPZ is formed in larger quantities in PZ alone compared to CESAR1. AMP does not form either MPZ or EPZ. Figure 5b–c shows MPZ formation as a function of the CO_2_ loading and its formation over time at different CO_2_ loadings. Higher temperatures favor MPZ formation and accumulation, and the concentrations observed seem to plateau after day 19. In Figure 5d–f, it can be seen that EPZ formation is slightly more temperature-dependent than MPZ formation and that EPZ concentrations increase over time for all three studied CO_2_ loadings. The drop in EPZ concentration from days 19 to 28 of thermal degradation was also observed in the experiment run at 150 °C but not at 120 °C under the same CO_2_ loading.

**Figure 5:**
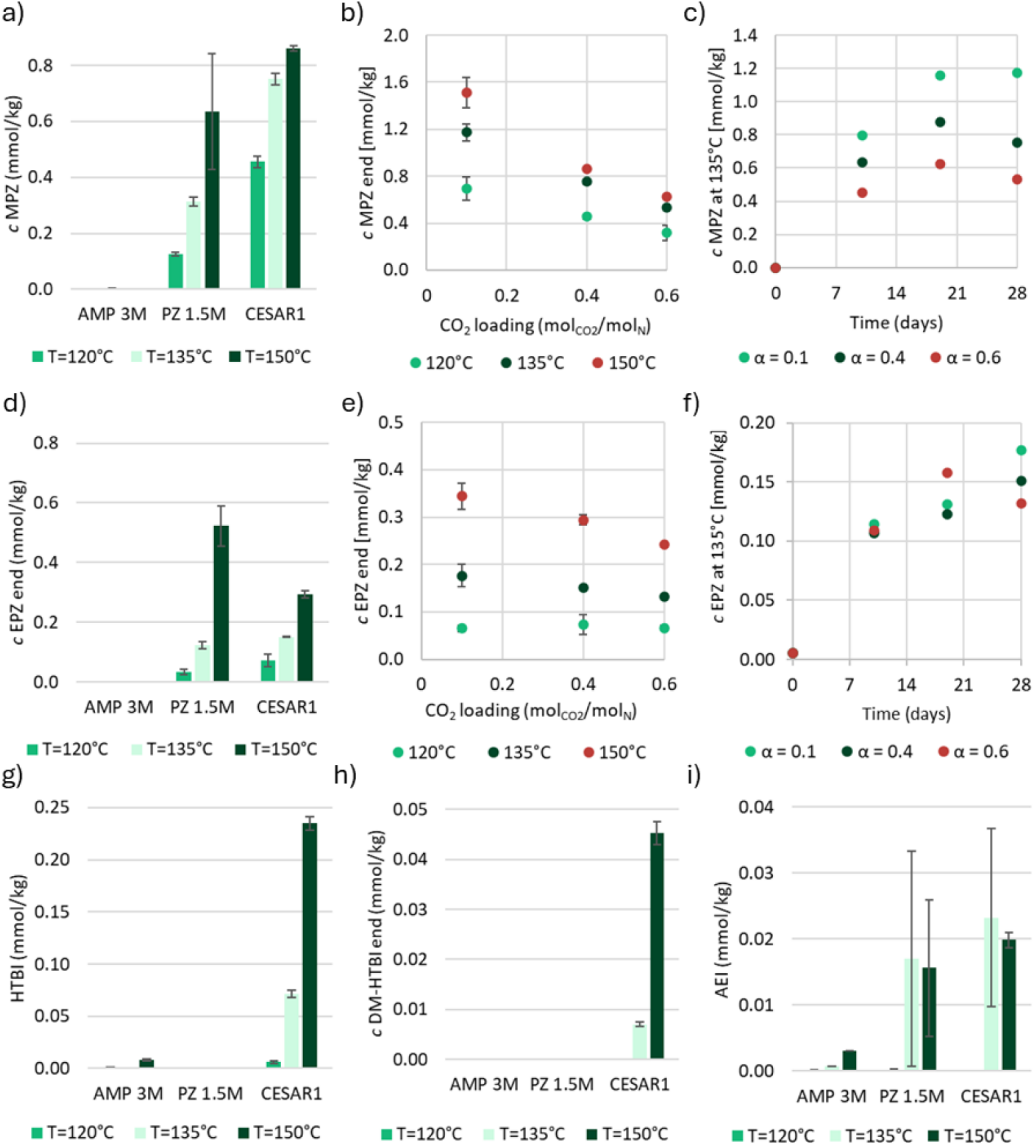
a) MPZ, d) EPZ, g) HTBI, h) DM-HTBI, and i) AEI concentrations in thermally degraded AMP, PZ, and CESAR1 solvents (α = 0.4 mol_CO2_/mol_N_) after 28 days at three different temperatures. b) MPZ and e) EPZ concentrations in the thermal degradation experiments with CESAR1 after 28 days, as a function of CO_2_ loading, and c) MPZ and f) EPZ as a function of time at 135 °C.

Three imidazolidinones, in addition to AEAEPZ-urea, were found in the degraded CESAR1 solvents after thermal degradation. The most abundant of these is HTBI, followed by DM-HTBI and AEI. Figure 5g–i shows their concentrations also in AMP and PZ alone, in addition to CESAR1 at three different temperatures. Both HTBI and DM-HTBI seem to form only in CESAR1, while AEI also forms in PZ alone.

HTBI clearly requires AMP to be present to form. Based on the suggested formation mechanisms of other cyclic ureas or imidazolidinones, the mechanism shown in Scheme 8 is proposed. The dimer of EDA and AMP, 2-[(2-aminoethyl)­amino]-2-methyl-1-propanol (AAMP), has been found in degraded CESAR1, predominantly after thermal degradation. This compound was detected after all other analyses for this work were completed and is therefore not quantified in all samples, but only in two available thermally and oxidatively degraded solvent samples. 0.14 mmol/kg of AAMP was found in thermally degraded CESAR1 (α = 0.4 at 150 °C, in the sample taken on day 19). Oxidatively degraded CESAR1 (after 5 weeks) contained only 0.002 mmol/kg of AAMP. The confirmed presence of AAMP in degraded CESAR1 strengthens the hypothesis of HTBI formation, which takes place according to Scheme 8.

**Scheme 8:**
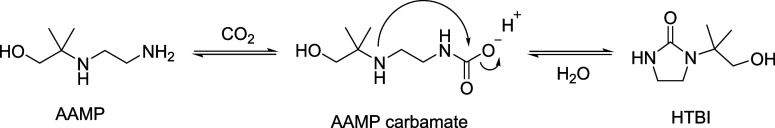
Suggested Mechanism for the Formation of HTBI from the Carbamate of AAMP

One suggested pathway for the formation of DM-HTBI is via the urea of AMPAMP, and an analogous intramolecular condensation reaction is shown in Scheme 9. The fact that this compound is present in such small amounts and only under the more heavily degrading conditions of CESAR1 at *T* ≥ 135 °C could be due to the larger steric hindrance in AMPAMP compared to AAMP, which probably forms HTBI.

**Scheme 9:**
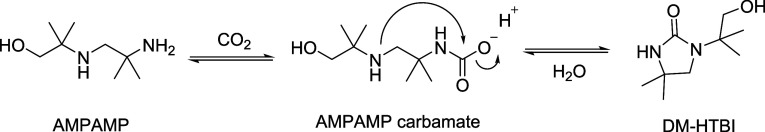
Suggested Mechanism for the Formation of DM-HTBI from AMPAMP Carbamate

AEI can be formed through a carbamate of diethylenetriamine (DETA), as illustrated in Scheme 10 and suggested by Freeman and Rochelle.^43^ AEI was identified as the main thermal degradation product of DETA^64^ and has also been seen in thermally degraded PZ.^43^ DETA itself has been proven to be very labile under thermal stress^64,67^ but is suspected to form a stable imidazolidinone in AEI.^4^ DETA was not found above the LOQ of 0.01 mmol/kg in the two samples in which AAMP was also quantified. This is in line with Freeman and Rochelle, who also found AEI but no DETA and hypothesized thermal decomposition of DETA upon formation.^43^

**Scheme 10:**
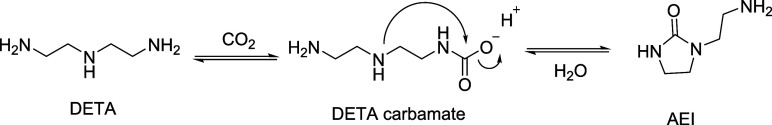
Suggested Reaction Scheme for the Formation of AEI via the Carbamate of Diethylenetriamine, as Proposed by Freeman and Rochelle^43^

A series of volatile degradation compounds was found to form during thermal degradation of AMP, PZ, and CESAR1, and the total concentrations of these at the end of the thermal degradation experiments are given in Figure 6. The compounds included are formaldehyde, acetaldehyde, acetone, ammonia, and six alkylamines. AMP produces 4–5 times more volatile degradation compounds than PZ under these conditions. At 120 °C, CESAR1 produces about the same amount of volatile degradation products as the sum of the individual amines, but at 135 and 150 °C, the concentrations in CESAR1 are much higher than in the single amines separately.

**Figure 6:**
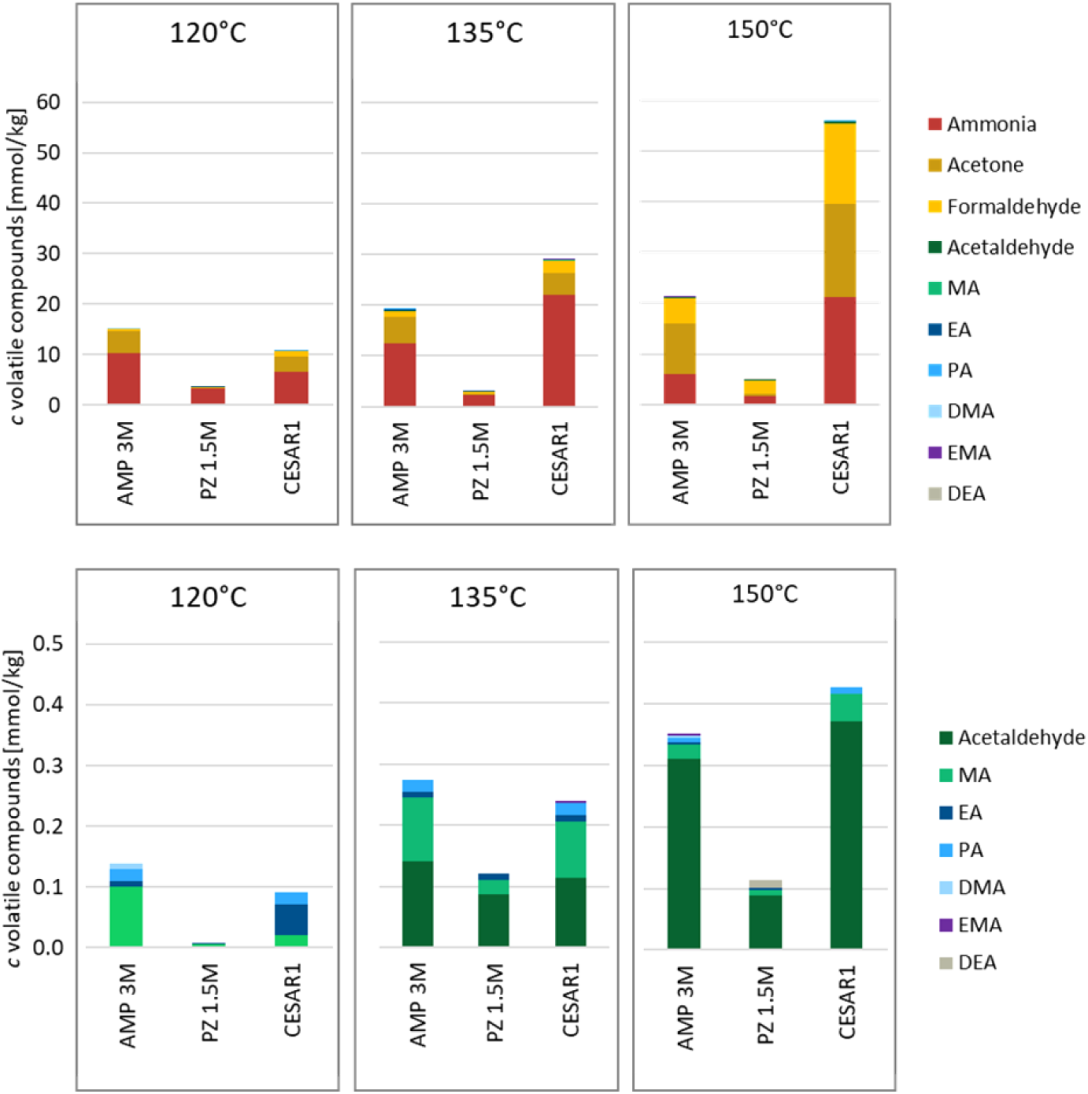
Volatile degradation compounds formed during thermal degradation for 28 days with α = 0.4 mol_CO2_/mol_N_. The bottom graphs show the same data as the top but the more abundant compounds are omitted to highlight concentration differences between the less abundant compounds.

As the concentrations of volatile compounds are very sensitive to sample handling, i.e., the temperature of the solvent when the thermal degradation cylinders were opened, the concentrations observed here should be considered semiquantitative.

The volatile degradation compounds consist mainly of ammonia and acetone, with some formaldehyde. The less abundant volatile degradation products are shown at the bottom of Figure 6, where it can be seen that acetaldehyde becomes more abundant with increasing temperature in all three solvents, and the alkylamine concentration is the highest at 120 °C. Additionally, a slight dependence on CO_2_ loading is found at 135–150 °C, where α = 0.4 mol_CO2_/mol_N_ gives the highest concentration of volatile degradation compounds, higher than both 0.1 and 0.6 mol_CO2_/mol_N_ (data are provided in the Supporting Information).

The thermal degradation tests in closed cylinders are not actual corrosion tests and cannot directly provide information about a solvent’s corrosivity. The concentration of dissolved metals at the end of the campaign can, however, be used as a corrosivity indicator.

Figure 7 shows the concentration of five stainless-steel elements dissolved in the AMP, PZ, and CESAR1 solvent samples with different CO_2_ loadings (α). Nickel (Ni) is the most abundant dissolved metal, followed by iron (Fe). The CESAR1 solvent, with the highest CO_2_ loading contains the highest concentration of Ni and also the highest sum of dissolved metals. The most degraded solvent samples, i.e., the ones, with the highest CO_2_ loadings and at the highest temperatures, have the highest dissolved metal concentrations. In comparison, thermal degradation tests of 30 wt % MEA (*aq.*) result in more than 4 times higher concentrations of dissolved metals in the solvent,^68^ indicating that CESAR1 is generally less corrosive than MEA.

**Figure 7:**
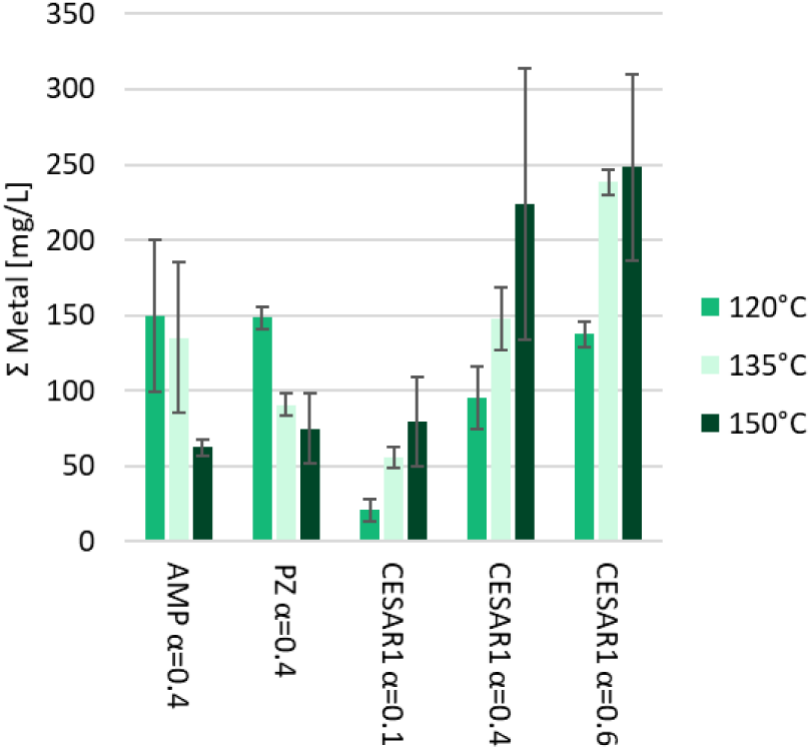
Concentration of dissolved stainless-steel metals in the thermally degraded solvent samples after 4 weeks. Error bars represent the standard deviation of the mean.

### 3.2 Oxidative Degradation of CESAR1

The difference in stability of the single amine solutions compared to that of CESAR1 becomes more evident during oxidative degradation than thermal degradation. The degradation rate of the CESAR1 blend gives about a 20% loss of both AMP and PZ, while no significant AMP loss could be determined when it was run under oxidizing conditions alone, and about 8% of PZ was lost during oxidative degradation of PZ. The higher stability of AMP compared to PZ is in line with the existing literature.^4,6,69^ No significant loss of MAMP was observed in any of the oxidative degradation experiments. Because of the inherent difficulty in accurately assessing the mass and water balance throughout these oxidative degradation experiments, the focus in this section will be on the formation of degradation compounds and not amine loss. The quantities of AMP and PZ throughout the experiments can be viewed in the Supporting Information.

The concentrations of all quantified oxidative degradation products in CESAR1, as well as in aqueous AMP and PZ separately, can be viewed in the comparison in Figure 8. Degradation in AMP and PZ is much lower than that in CESAR1, and in most cases, the concentrations of degradation products are so much lower than in CESAR1 that they can barely be seen in Figure 8. Altogether, 34 different degradation compounds were found in the oxidatively degraded CESAR1 solvent. The main product of CESAR1 degradation under the studied conditions for oxidative degradation was FAc, which also forms in AMP and PZ separately. FAc is also known to be one of the most abundant degradation products in other comparable experiments with other amines in the same or similar types of setups.^6,60^ Of the 34 compounds found above the LOQ in oxidatively degraded CESAR1, six compounds have not previously been quantified in PZ, AMP, or any blend of the two in the open literature. These compounds are HMeGly, F-AMP, AEAA, AIBA, AEHA, and NMAMP.

**Figure 8:**
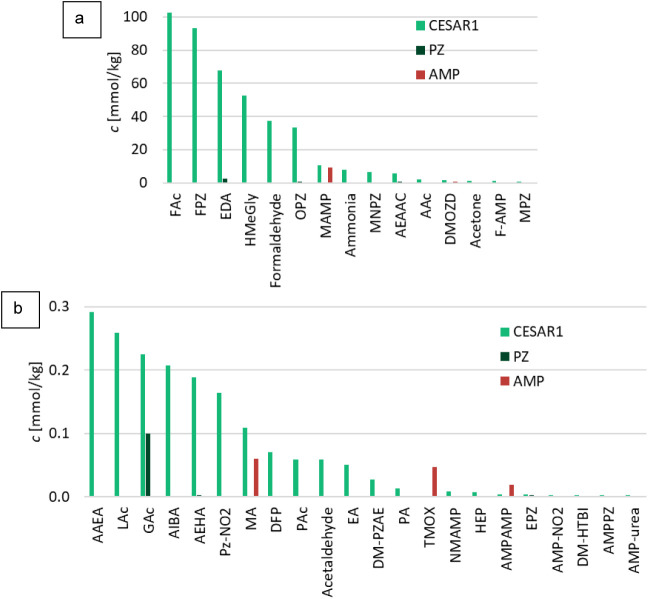
Concentrations of all degradation products (and MAMP) in the CESAR1, PZ, and AMP solvents after 6 weeks of oxidation at 60 °C. The compounds in (b) are not shown in (a) due to their comparatively low concentrations and vice versa.

Only 15 degradation compounds were found in the oxidatively degraded single amine solvents of PZ and AMP. From Figure 9, it is evident that eight of the degradation compounds, which are also found in CESAR1, seem to originate from PZ, while six originate from AMP. Only FAc forms during oxidative degradation of both AMP and PZ. The formation mechanisms of the known and frequently studied degradation compounds of PZ and AMP; EDA, FPZ, OPZ, and F-AMP will not be discussed in this work, as they are abundantly investigated and described in the literature.^30,31,38,43^

**Figure 9:**
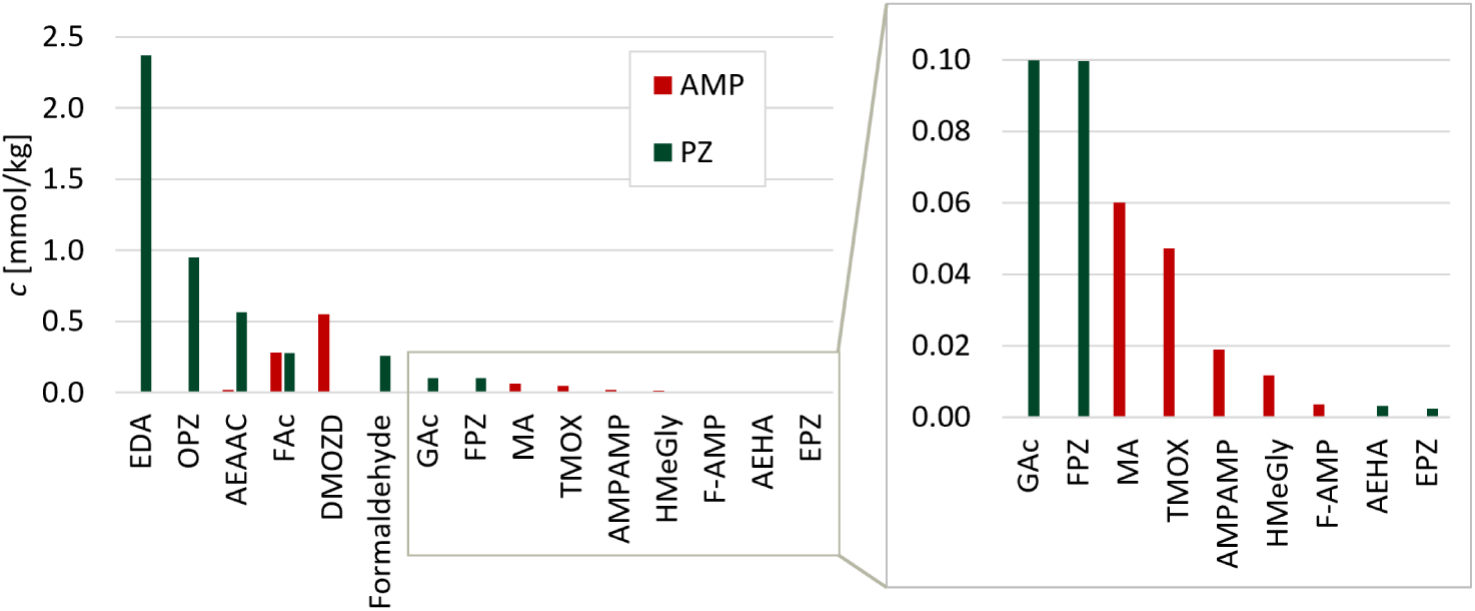
Concentrations of all quantified oxidative degradation products in the single component experiments (aqueous AMP 3 M and aqueous PZ 1.5M).

The fact that 19 of the compounds found in oxidatively degraded CESAR1 do not appear in oxidatively degraded PZ or AMP is likely due to two reasons. First, the degradation rate of the blend is much higher than that of the single amines. Second, some degradation compounds require the presence of both AMP and PZ to form.

HMeGly barely forms in AMP alone but is the fourth most abundant compound in oxidatively degraded CESAR1. This could be solely an effect of the higher degradation rate of CESAR1 compared to that of AMP alone, or it could be that the formation of an intermediate of HMeGly occurs to a larger extent during PZ degradation, allowing for a higher abundance of HMeGly in the blend. Figure 10 shows that the HMeGly concentration during AMP oxidation is barely above the LOQ, while the formation or accumulation rate of HMeGly is steady in CESAR1.

**Figure 10:**
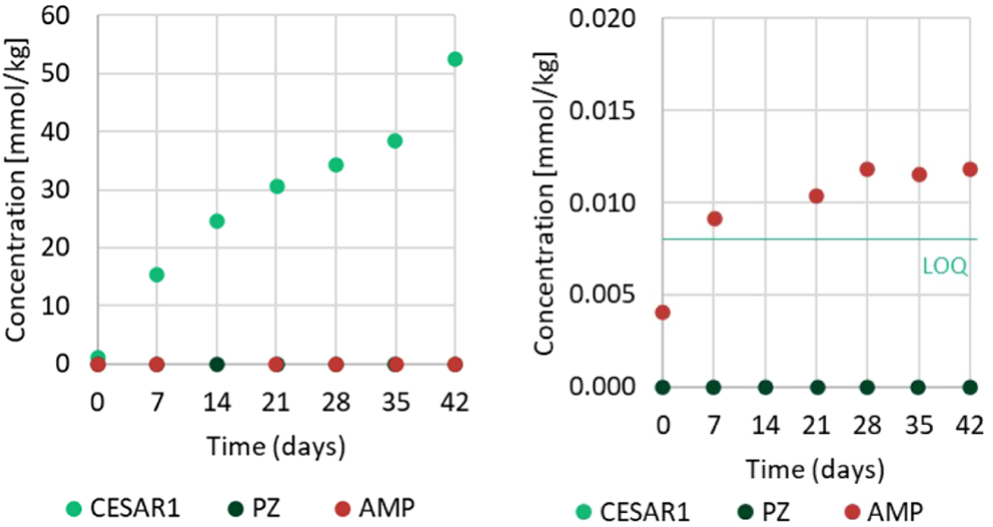
HMeGly concentration during oxidative degradation of CESAR1, PZ, and AMP.

HMeGly is an analogous degradation compound to *N*-(2-hydroxyethyl)-glycine (HEGly) formed in MEA, which occurs during CESAR1 degradation. HMeGly contains an AMP moiety instead of the MEA moiety in HEGly, explaining why it does not form in PZ alone. The formation mechanism of HEGly has been hypothesized to take place either through an acid-catalyzed condensation reaction with glyoxylic acid^70^ or through intermediate formation of another degradation compound, *N*-(2-hydroxyethyl)-2-[(2-hydroxyethyl)­amino]-acetamide (HEHEAA).^25^ The high abundance of HMeGly in CESAR1 could therefore possibly be explained if glyoxylic acid is formed to a larger extent in PZ than in AMP. Glyoxylic acid was not quantified in this work, but based on the molecular structure of PZ, which does contain ethyl chains, it is more plausible for glyoxylic acid to form through PZ degradation than during degradation of AMP, which has a branched propyl chain. AEAAC, which is found in oxidatively degraded PZ and CESAR1, is likely formed through the same mechanism as HMeGly, with EDA as a substrate instead of AMP.

AAEA is not found in PZ and AMP alone but is present in oxidatively degraded CESAR1, albeit in low concentrations. Despite not being present in PZ after 6 weeks under oxidizing conditions, the most likely formation pathway of AAEA is through the PZ degradation compounds glycine and EDA, according to Scheme 11. Glycine is a known degradation compound of PZ,^31,40,71^ which was not studied in this work due to the difficulty of analyzing glycine with our in-house LCMS equipment and methodology (i.e., its tendency to be overestimated, as HMeGly partly fragments and forms some glycine in the ion source).

**Scheme 11:**
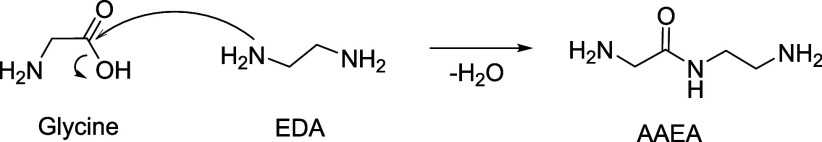
Formation Pathways for AAEA from EDA and Glycine, as Suggested by Wang^31^

The presence of MA, which was found in oxidatively degraded AMP but not in PZ, could explain why MPZ is found only in CESAR1 and not in PZ under these conditions. EPZ is found in comparable abundances in oxidatively degraded PZ and CESAR1. As for the formation of AAEA in CESAR1 and not in PZ, we hypothesize that the increased PZ degradation in the presence of AMP causes further degradation and additional pathways to take place under the otherwise same conditions, which is the cause of MPZ only being found in the CESAR1 and not in the PZ solvent.

AEHA was suggested to be formed as an intermediary product during PZ degradation by Wang^31^ on the pathway to OPZ formation. The suggested reaction mechanism is shown in Scheme 12. Only traces of AEHA were found in the oxidatively degraded PZ, but during oxidative degradation of CESAR1, a steadily increasing concentration of AEHA was observed throughout the 6 weeks. An analogous compound, 2-hydroxy-*N*-(2-hydroxyethyl)­acetamide (HHEA), is found during MEA degradation; however, only in very low concentrations.^72^ The reason for AEHA being more abundant during CESAR1 degradation than HHEA in MEA is likely due to the higher formation of GAc in PZ/CESAR1 compared to MEA.

**Scheme 12:**
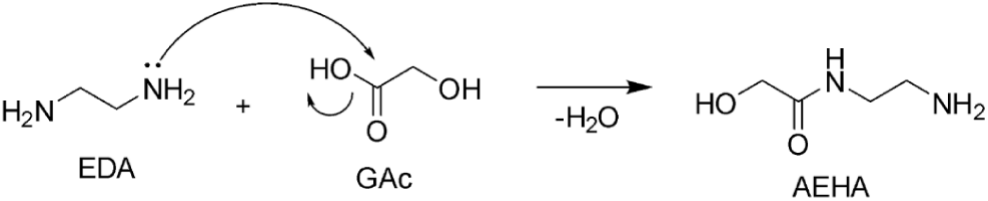
Formation Mechanism of AEHA through Condensation of EDA and GAc, as Suggested by Wang^31^

Organic acids, or carboxylates, are common oxidation products of amines, as they are for other organic molecules. The main acid formed during CESAR1 oxidation is FAc, which is also the case for AMP and PZ. The second most abundant acids are AAc and LAc, which are not present in the single amines alone. LAc has, in fact, not yet been reported as a product of degradation of any of these amines in the literature but was found in CESAR1 used for pilot operation at TCM.^28^ GAc is a product of PZ oxidation but is much more abundant in oxidatively degraded CESAR1 than in PZ alone. PAc is the last organic acid present in quantifiable amounts in degraded CESAR1. PAc has also been quantified in CESAR1 previously during piloting at Niederaussem and TCM.^7,28,73^

AIBA is another amino acid found in oxidized CESAR1, which can be formed through direct oxidation of the carbon holding the hydroxyl group of AMP, as illustrated in Scheme 13.

**Scheme 13:**
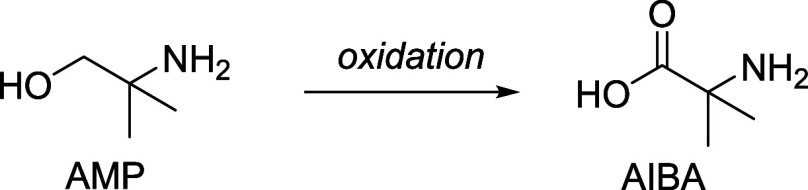
Oxidation of AMP to Form AIBA

#### 3.2.1 Nitrosamine Formation in CESAR1

Even during oxidative degradation with only compressed air, as was performed in this work, we observe the formation of nitrosamines and nitramines in CESAR1. Figure 8a shows that MNPZ is even among the ten most abundant degradation compounds after oxidative CESAR1 degradation. Since the oxidative degradation experiments were performed using compressed air, trace amounts of NO_*X*_ corresponding to the local ambient air concentration are expected to be present in the gas phase. With local NO_2_ concentrations in Trondheim averaging 19 μg/m^3^, and NO at 14 μg/m^3^, this does not suffice to explain the observed concentrations in the CESAR1 solvent, meaning that reaction pathways are taking place other than the direct nitrosation by the gas phase or dissolved NO_2_. In Figure 11a, it can be seen that the concentrations of both MNPZ and NMAMP steadily increase over time during the degradation experiment. Nielsen et al.^44^ also observed the formation of a small amount of MNPZ (up to 0.09 mmol/kg) in a laboratory test of PZ with both absorption and stripping cycles, using a synthetic gas consisting of air and CO_2_. They hypothesized that some of the PZ degrades to form nitrite under absorber conditions, which then acts as the nitrosation agent under stripper conditions. Nitrite formation has been observed in the same setup during oxidative degradation of other amines.^60^ This observation also aligns with the observations made at RWE’s PCC pilot plant at Niederaussem, where NO_2_ removal was tested as a solvent management strategy without giving any significant decrease in the nitrosamine concentrations in the solvent.^7^ Nitrite and nitrate could not be studied with the analytical techniques available in this work.

**Figure 11:**
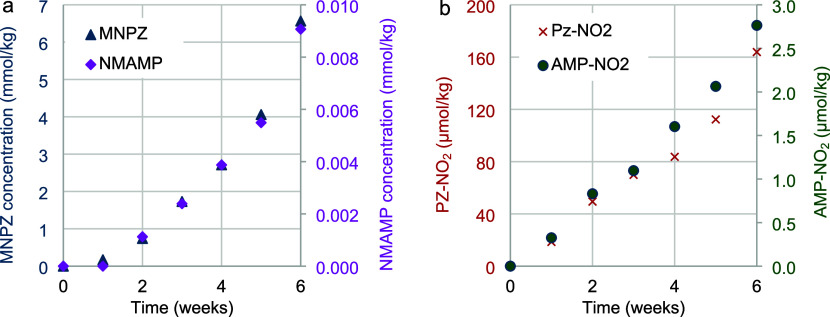
Concentration of a) nitrosamines and b) nitramines in CESAR1 during oxidative degradation.

Even the two nitramines PZ-NO_2_ and AMP-NO_2_ are seen to steadily increase in concentration during oxidative CESAR1 degradation, as shown in Figure 11b.

#### 3.2.2 Volatile Degradation Compounds

As mentioned earlier, each oxidation experiment was equipped with two acid wash solutions of 0.1 M H_2_SO_4_ in series. These acid wash solutions were analyzed for all the studied compounds in this work to assess which solvent components are likely to appear in the emissions from the CO_2_ capture process with CESAR1. The amount of a compound captured in the two washes over a six-week experiment is summed up and shown in Figure 12. Ammonia is by far the most dominant compound to get emitted from the oxidation experiments and accumulated in the acid washes, as can be seen in Figure 12a. The CESAR1 blend gave orders of magnitude higher amounts of NH_3_ in the acid washes compared to the single amines, where the AMP experiment gave a total of 0.1 mmol and the PZ experiment gave 0.5 mmol of NH_3_, which is not possible to see on the same scale as CESAR1 in this graph. The solvent amines themselves are not emitted and collected in very large amounts, and Figure 12b shows that solvent amine emissions are only slightly higher in the blend than in AMP and PZ separately. Notably, the PZ emissions seem to decrease when PZ is blended with AMP, while the AMP emissions from CESAR1 are slightly higher than they are from AMP alone.

**Figure 12:**
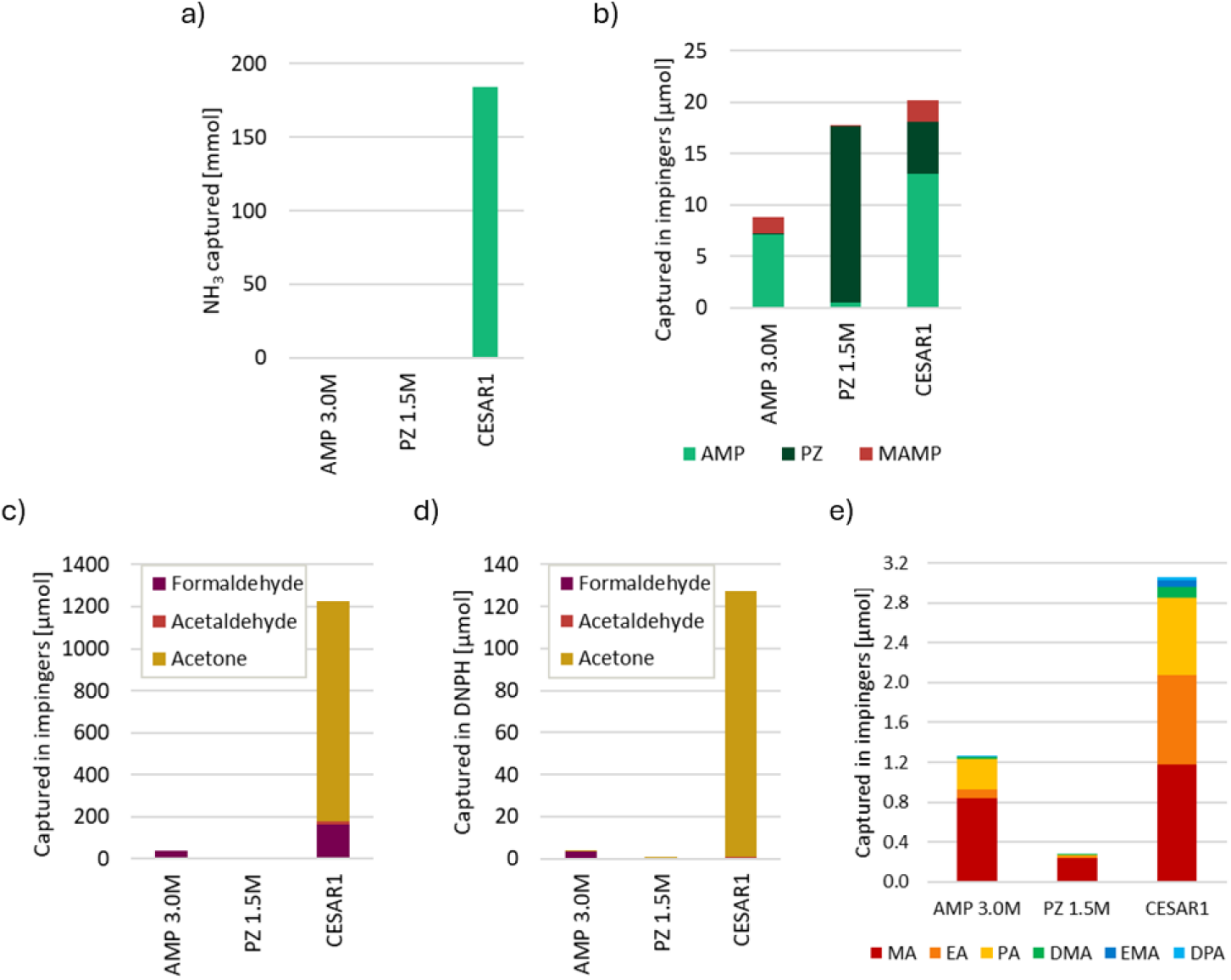
Absolute amounts of a) ammonia and b) solvent amines and MAMP captured in the acid washes during oxidative degradation of AMP, PZ, and CESAR1; amount of acetone, formaldehyde, and acetaldehyde accumulated c) in the impinger bottles throughout the 6 weeks and d) DNPH-cartridge attached to the gas bleed during the last 4 days of each oxidative degradation experiment; e) total amount of alkylamines captured in the acid washes during oxidative degradation.

Also, the carboxylates formaldehyde, acetaldehyde, and acetone in Figure 12c–d are much more abundant during CESAR1 degradation than in the individual amines, where especially the acetone formation and emissions are significantly higher in the blend than in, i.e., AMP. Formaldehyde emissions clearly originate from AMP degradation under oxidative conditions, as PZ produces very little formaldehyde, which is evident from both the contents of the acid washes and the DNPH cartridges. Acetaldehyde emissions are low in all three systems. Analysis of the acid washes from the three systems showed quantifiable amounts of acetaldehyde and acetone only from CESAR1.

Alkylamines are produced during the oxidation of AMP and PZ, as shown in Figure 12e. Oxidative degradation seems to predominantly produce methylamine (MA) emissions, but larger alkylamines are also present in the acid washes. Notably, propylamine (PA) is a product of AMP oxidation. The concentrations of all alkylamines except MA increase with the blend compared to the two single amines combined.

Other compounds that were also found in the acid wash solutions from CESAR1 degradation in significant amounts (more than 0.5 μmol) were EDA, MAMP, and HMeGly.

Due to the nature of the thermal degradation experiments, a similar evaluation could not be performed in those experiments.

### 3.3 Nitrogen Balance

Total nitrogen (TN) analysis of the samples is performed to check whether all major degradation compounds in the solvent have been identified with the existing methodology. The nitrogen balance (recovery) is calculated as the molar ratio between nitrogen from all compounds determined by LC-MS and the individual total nitrogen (TN) analysis, as given in eq 2.

2 $$R_{N}=\frac{\overset{n}{\underset{i=1}{\sum }}c_{i}^{\mathrm{LC‐MS}}}{c_{TN}}$$

From analytical uncertainties (3% relative for the solvent amines and 5% relative for TN), the relative uncertainty of *R*_N_ is estimated to be 6%.

The nitrogen balances for the end samples of the oxidative experiments are summarized in Figure 13a. The recoveries are in the range from 97 to 105%, which means that the nitrogen balance is closed when uncertainties are taken into consideration.

**Figure 13:**
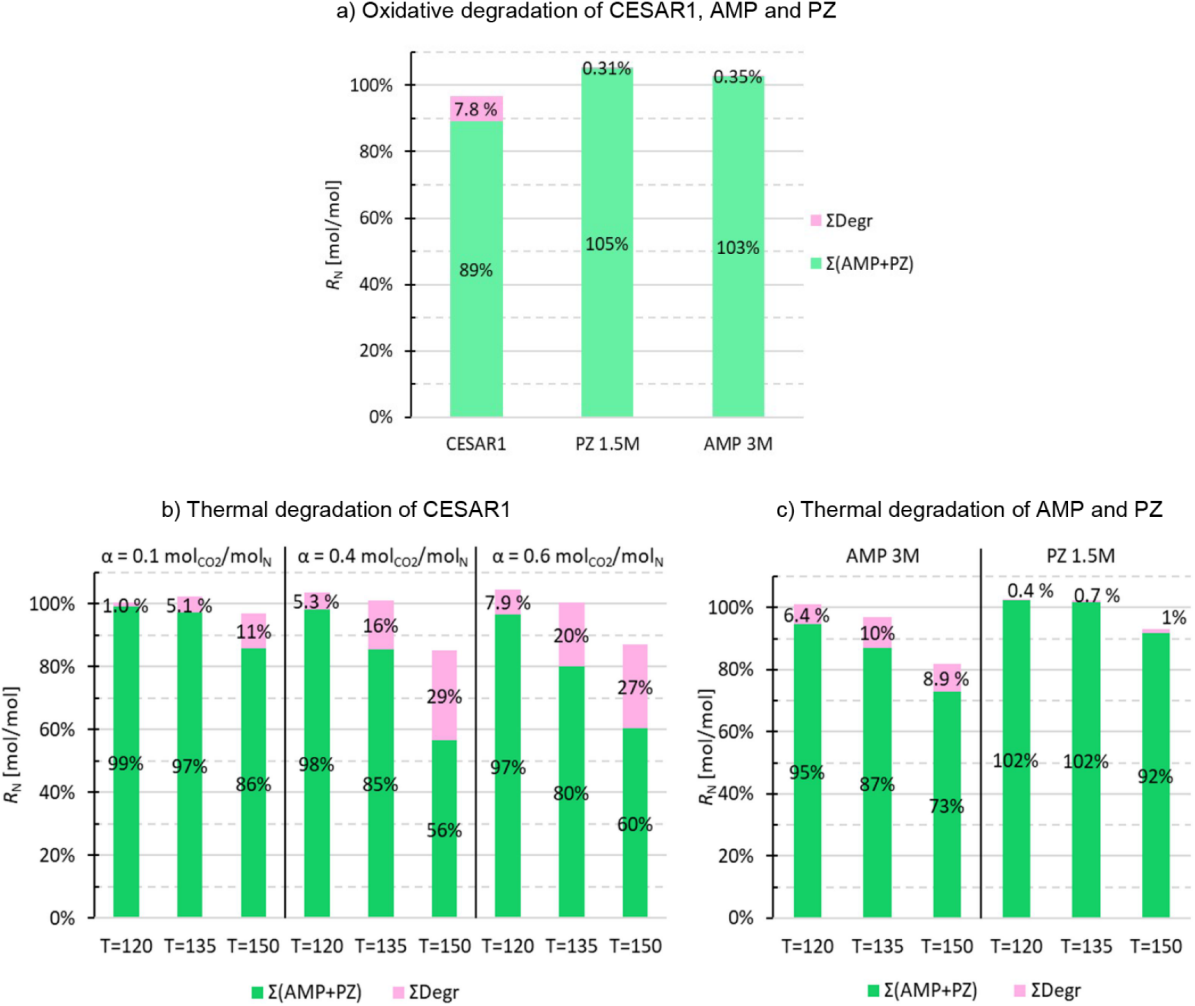
a) Nitrogen balance end samples of oxidative degradation experiments; b) nitrogen balance end samples of thermal degradation with CESAR1, here loading (α) and temperature (*T*) used are indicated on the *x*-axis; c) nitrogen balance end samples of thermal degradation experiments with 3 M AMP and 1.5 M PZ on the right, here temperature (*T*) used are indicated on the *x*-axis (all with loading, α = 0.4 mol_CO2_/mol_N_).

The nitrogen balances for the end samples of the thermal degradation experiments are summarized in Figure 13b–c. Here, the recoveries are in the range of 85–105% for the experiments with CESAR1, with only two below 94%, meaning their nitrogen balance was not closed. These were samples degraded at 150 °C with α = 0.4 (*R*_N_ = 85%) and 0.6 mol_CO2_/mol_N_ (*R*_N_ = 87%), respectively. The recoveries for the thermal degradation experiments with 3 M AMP are in the range of 82–101%. Also, in this case, it is only the one at 150 °C that is below 94% (*R*_N_ = 82%). The same is also true with 1.5 M PZ, where the experiments at 150 °C have a recovery of 93%, and the two others are in the range of 102% and 103%. This means that all of the recoveries from thermal degradation experiments (end samples), except some at 150 °C, have a closed nitrogen balance within the analytical uncertainties. As the conditions in the thermal degradation tests at 150 °C are quite tough and not expected to be representative of what occurs under normal process conditions, this is very satisfactory, although it is evident that some thermal degradation compounds exist which were not studied in this work. Though the nitrogen balances are considered closed when analytical uncertainties are considered, there could still be minor degradation compounds that are not identified in the degraded solvent samples.

## 4 Conclusions

A total of 48 degradation compounds were quantified in CESAR1, and new analytical methods were developed for many of these. Of these 48 degradation compounds, 37 were found to form under oxidizing conditions, three of which were only found in the gaseous emissions from the solvent. 37 compounds were found to form under thermal stress. Six of the oxidative and seven thermal degradation compounds from this work have not previously been quantified in the CESAR1 solvent in the open literature. Additionally, two organic acids were formed during thermal degradation, which have not been previously found in CESAR1.

The findings show that the most abundant nitrogen-containing degradation products of CESAR1 have been identified and quantified. There is, however, reason to believe that the degraded solvent contains some additional species that were not included in this study. Most of these unknown compounds are products of thermal degradation, which is less dominant during pilot or large-scale CO_2_ capture than oxidative degradation. Thermal degradation will be more relevant, i.e., during thermal reclaiming or if high-temperature regeneration is applied. During oxidative degradation, the nitrogen balance is closed within the analytical uncertainty, but it is possible that some less abundant CESAR1 degradation compounds remain unidentified in this work.

CESAR1 forms nitrosamines and nitramines, even in the absence of NO_2_ (or NO_*x*_). This means that NO_*x*_ removal from the flue gas during CO_2_ capture may not guarantee a low concentration or absence of nitrosamines in the solvent. It would, therefore, be important to monitor nitrosamines and nitramines in the process and ensure that these do not reach the capture plant environment. Furthermore, this finding highlights the need for further study of the mechanisms of formation of nitrosamines and nitramines in the postcombustion CO_2_ capture process.

CESAR1 degrades more rapidly than the sum of the two individual amines separately. CESAR1 forms both compounds found in the individual amines, AMP and PZ, but also compounds that require both amines to be present. CESAR1 also forms more volatile degradation compounds, such as formaldehyde, acetaldehyde, ammonia, and alkylamines, than the individual components. Monitoring of volatile degradation compounds is therefore advisable both in the cleaned flue gas and potentially the CO_2_ product.

## Supplementary Material



## Notes

The total concentration of each individual stainless-steel metal measured by ICP-MS after thermal degradation (Figure S1); the data from all thermal degradation experiments (Tables S1–S8); the detailed results from the analysis of acid washes (Tables S9–S12); the solvent after, or during oxidative degradation of the three different solvents (Tables S13–S16) (PDF)

## Abbreviations

*aq.*: aqueous
DNPH: 2,4-dinitrophenylhydrazine
LC: liquid chromatography
LOD: limit of detection
LOQ: limit of quantification
M: mol/kg
MEA: monoethanolamine
MS: mass spectrometry
NDIR: nondispersive infrared
NO_X_: nitrogen oxides
PCC: postcombustion capture
TCM: Technology Centre Mongstad
TIC: total inorganic carbon
TN: total nitrogen
TOC: total organic carbon
