# Supplementary material for: Degradation of 2‑Amino-2-methylpropanol and Piperazine at CO2 Capture-Relevant Conditions
Source: Ind Eng Chem Res. 2025 May 23;64(22):11000–20. doi: 10.1021/acs.iecr.5c00527 (PMC12142809; doi:10.1021/acs.iecr.5c00527)
Supplement: Supplementary file 1 [file ie5c00527_si_001.pdf]

Supporting information:

Degradation of 2-amino-2-methylpropanol and piperazine at CO_2 capture relevant conditions

Vanja Buvik[1]*, Kai Vernstad[1], Andreas Grimstvedt[1], Karen K. Høisæter[2], Solrun J. Vevelstad[1], Hanna K. Knuutila[3]

[1] SINTEF Industry, 7465 Trondheim, Norway
[2] Technology Centre Mongstad, 5954 Mongstad, Norway
[3] Department of Chemical Engineering, NTNU, 7491 Trondheim, Norway

* Corresponding author: vanja.buvik@sintef.no

Figure S 1 shows the total concentration of each individual stainless-steel metal measured by ICP-MS after thermal degradation. Table S 1-8 show the data from all thermal degradation experiments, while Table S 9-12 show the detailed results from analysis of acid washes and Table S 13-16 the solvent after, or during oxidative degradation of the three different solvents.

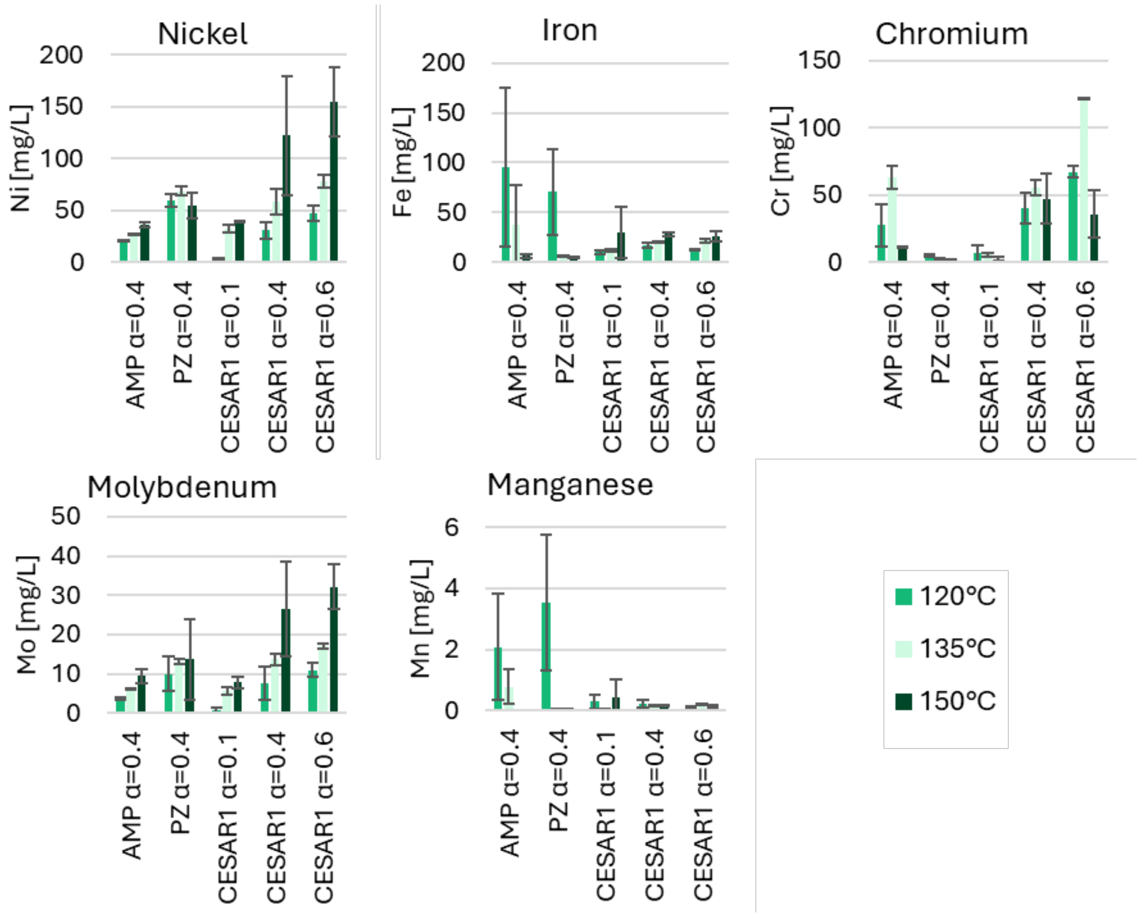

Figure S 1: Measured concentrations of all individual metals after thermal degradation of AMP, PZ and CESAR1 in metal cylinders.

Table S 1: Measured concentrations in thermally degraded AMP 3M, PZ 1.5M and CESAR1 in mg/kg, table 1 of 8.

	Acetone			AEAAC			AEI			AEAEPZ			AEAEPZ urea		
	120°C	135°C	150°C	120°C	135°C	150°C	120°C	135°C	150°C	120°C	135°C	150°C	120°C	135°C	150°C
AMP 3M, α=0.4, D28	245	298	378	< 1	< 1	< 1	0.02	< 0.1	0.4	< 1	< 0.1	< 0.1	1.1	< 0.1	< 0.1
AMP 3M, α=0.4, D28	244	301	514	< 1	< 1	< 1	0.02	0.1	< 0,1	< 1	< 0.1	< 0.1	0.7	< 0.1	< 0.1
PZ 1.5M, α=0.4, D28	< 50	18	20	70	98	18	0.03	0.1	3.3	3.1	5.7	2.5	1.7	23.6	199.1
PZ 1.5M, α=0.4, D28	< 50	16	16	69	94	34	0.02	4.3	0.7	2.4	7.7	1.5	2.9	25.7	194.3
CESAR1, α=0.1, D10	156	163	339	26	75	125	-	< 0.1	0.2	3.2	22.7	29.1	0.2	2.8	35.2
CESAR1, α=0.1, D19	284	225	285	25	68	110	-	< 0.1	0.5	7.2	41.7	42.1	0.4	12.8	118.8
CESAR1, α=0.1, D28	153	284	442	20	107	93	-	1.2	0.9	11.0	59.1	52.9	1.2	33.4	211.8
CESAR1, α=0.1, D28	145	235	325	24	97	106	0.001	0.6	1.1	8.2	51.5	45.2	0.6	21.5	187.1
CESAR1, α=0.4, D10	162	251	585	15	44	39	0.02	1.9	0.4	3.5	18.0	13.5	0.8	8.4	62.8
CESAR1, α=0.4, D19	168	212	710	17	38	21	-	< 0.1	1.4	4.6	22.2	10.5	0.9	24.0	107.6
CESAR1, α=0.4, D28	182	257	867	24	31	18	0.004	4.7	2.4	7.6	24.3	20.1	2.1	42.9	145.6
CESAR1, α=0.4, D28	168	245	794	16	38	12	-	1.3	2.7	7.2	24.7	12.8	2.2	45.2	153.2
CESAR1, α=0.6, D10	126	146	431	6.5	13	< 1	-	0.4	1.4	2.2	12.8	10.0	0.3	10.6	61.9
CESAR1, α=0.6, D19	114	173	675	12	13	< 1	-	0.1	2.1	3.6	14.0	7.2	1.0	28.7	110.5
CESAR1, α=0.6, D28	110	136	525	14	13	< 1	0.02	2.3	2.3	5.2	15.1	7.5	1.7	40.8	91.0
CESAR1, α=0.6, D28	114	150	-	12	9.3		0.07	0.2		4.5	13.3		1.9	41.1	

Table S 2: Measured concentrations in thermally degraded AMP 3M, PZ 1.5M and CESAR1 in mg/kg, table 2 of 8.

	AMPAMP			AMP urea			DMOZD			DM-PZEA			DM-HTBI		
	120°C	135°C	150°C	120°C	135°C	150°C	120°C	135°C	150°C	120°C	135°C	150°C	120°C	135°C	150°C
AMP 3M. α=0.4. D28	974	3185	3057	14.6	30.4	14.6	16427	24098	23954	< 0.1	3.0	2	< 0.1	< 0.1	< 0.1
AMP 3M. α=0.4. D28	908	2909	3012	14.7	29.5	14.7	16879	24373	23933	< 0.1	0.8	1	< 0.1	< 0.1	< 0.1
PZ 1.5M. α=0.4. D28	< 0.1	< 0.1	< 0.1	< 0.1	< 0.1	< 0.1	< 1	< 1	8	< 0.1	0.9	32	< 0.1	< 0.1	< 0.1
PZ 1.5M. α=0.4. D28	< 0.1	< 0.1	< 0.1	< 0.1	< 0.1	< 0.1	< 1	< 1	3	< 0.1	1.2	8	< 0.1	< 0.1	< 0.1
CESAR1. α=0.1. D10	69	411	1120	2.0	5.9	2.0	1270	2405	3566	245	2441	8256	< 0.1	< 0.1	0.36
CESAR1. α=0.1. D19	161	974	1963	2.5	6.2	2.5	1441	2609	3816	749	6391	18791	< 0.1	0.13	1.4
CESAR1. α=0.1. D28	287	1637	2331	2.6	6.7	2.6	1566	2723	3777	1187	12989	28522	< 0.1	0.46	3.2
CESAR1. α=0.1. D28	203	1353	2344	2.4	6.2	2.4	1363	2558	3836	956	8823	25965	< 0.1	0.29	2.6
CESAR1. α=0.4. D10	215	1636	1736	11.3	30.4	11.3	10915	23485	28237	963	14889	36296	< 0.1	0.30	2.4
CESAR1. α=0.4. D19	590	2156	1369	14.6	29.0	14.6	14553	23078	24590	2717	19206	54453	< 0.1	0.68	5.8
CESAR1. α=0.4. D28	782	2156	1039	15.4	27.7	15.4	14781	22720	21788	5426	28442	62682	< 0.1	1.2	7.8
CESAR1. α=0.4. D28	822	2279	1067	15.6	28.7	15.6	15961	21966	22382	5976	28371	64949	< 0.1	1.4	8.7
CESAR1. α=0.6. D10	227	1410	1229	10.4	32.0	10.4	19563	39998	36960	1646	12141	40907	< 0.1	0.49	3.5
CESAR1. α=0.6. D19	453	1483	812	14.1	33.6	14.1	27182	39435	30488	4348	24890	59090	< 0.1	1.3	8.8
CESAR1. α=0.6. D28	508	1512	962	14.2	33.6	14.2	27975	35747	31685	5641	31806	47908	< 0.1	1.6	6.7
CESAR1. α=0.6. D28	547	1401		14.9	34.1	14.9	27917	36488		6320	31583		< 0.1	1.5	

Table S 3: Measured concentrations in thermally degraded AMP 3M, PZ 1.5M and CESAR1 in mg/kg, table 3 of 8.

	EDA			EPZ			FPZ			HMeGly			HTBI		
	120°C	135°C	150°C	120°C	135°C	150°C	120°C	135°C	150°C	120°C	135°C	150°C	120°C	135°C	150°C
AMP 3M, α=0.4, D28	< 10	< 1	< 1	< 0.1	< 0.1	< 0.1	< 1	< 1	< 0.1	14	17	8	0.2	< 1	1.3
AMP 3M, α=0.4, D28	< 10	< 1	< 1	< 0.1	< 0.1	< 0.1	< 1	< 1	< 0.1	33	22	19	0.1	< 1	1.0
PZ 1.5M, α=0.4, D28	95	153	197	2.8	12.9	67.3	82	497	1359	< 1	< 1	< 1	< 0.1	< 1	< 0,1
PZ 1.5M, α=0.4, D28	99	157	175	5.0	15.6	52.1	135	264	700	< 1	< 1	< 1	< 0.1	< 1	< 0,1
CESAR1, α=0.1, D10	86	176	136	4.7	13.0	16.0	46	108	124	346	511	383	< 0.1	1.1	8.3
CESAR1, α=0.1, D19	88	125	129	7.5	15.0	23.9	50	136	221	336	397	288	0.1	3.6	23
CESAR1, α=0.1, D28	76	138	208	8.2	22.9	42.4	56	174	267	298	333	264	0.4	9.5	40
CESAR1, α=0.1, D28	105	172	176	6.7	17.5	36.2	39	192	293	385	709	327	0.3	6.4	33
CESAR1, α=0.4, D10	182	224	210	3.9	12.2	18.0	53	198	600	432	466	179	0.2	2.8	14
CESAR1, α=0.4, D19	127	206	215	5.1	14.0	27.6	71	304	941	287	350	134	0.3	6.3	24
CESAR1, α=0.4, D28	146	222	245	10.7	17.0	32.3	69	379	943	301	243	120	1.1	10	32
CESAR1, α=0.4, D28	127	233	241	6.0	17.4	34.9	66	483	1055	260	283	120	0.6	11	34
CESAR1, α=0.6, D10	149	188	183	3.4	12.4	22.8	52	508	959	254	208	81	0.1	2.8	14
CESAR1, α=0.6, D19	124	210	194	6.0	18.0	31.0	160	760	1832	186	154	92	0.3	6.3	25
CESAR1, α=0.6, D28	114	249	197	7.0	15.3	27.6	118	857	1428	152	133	81	0.5	7.8	22
CESAR1, α=0.6, D28	109	203		8.2	14.8		208	662		133	98		0.4	7.5	

Table S 4: Measured concentrations in thermally degraded AMP 3M, PZ 1.5M and CESAR1 in mg/kg, table 4 of 8.

	HEP			PEP			NH3			MAMP			MPZ		
	120°C	135°C	150°C	120°C	135°C	150°C	120°C	135°C	150°C	120°C	135°C	150°C	120°C	135°C	150°C
AMP 3M, α=0.4, D28	< 0.1	< 0.1	< 0,1	< 0.1	< 0.1	< 0,1	161	258	603	166	138	80	< 0.1	0.6	0.2
AMP 3M, α=0.4, D28	< 0.1	< 0.1	< 0,1	< 0.1	< 0.1	< 0,1	191	167	794	165	141	88	< 0.1	0.5	0.3
PZ 1.5M, α=0.4, D28	< 0.1	0.8	8.72	2.7	30.2	152	81	38	316	< 1	< 0,1	0	13	33	84
PZ 1.5M, α=0.4, D28	< 0.1	1.9	2.55	4.7	21.1	107	36	40	95	< 1	< 0,1	0	12	30	43
CESAR1, α=0.1, D10	< 0.1	0.1	0.26	0.4	1.7	9.1	104	92	227	639	676	285	65	80	99
CESAR1, α=0.1, D19	< 0.1	0.1	0.66	0.5	2.5	16.1	200	167	323	557	515	241	75	116	120
CESAR1, α=0.1, D28	< 0.1	0.7	0.91	0.7	7.7	32.5	116	197	588	507	460	207	79	125	164
CESAR1, α=0.1, D28	< 0.1	0.6	0.91	0.5	4.7	24.3	124	181	524	544	502	215	60	110	139
CESAR1, α=0.4, D10	0.004	1.4	0.60	0.2	1.8	8.0	104	162	576	362	153	60	37	63	68
CESAR1, α=0.4, D19	< 0.1	0.5	1.21	0.2	3.6	17.4	95	208	1383	228	120	55	62	88	74
CESAR1, α=0.4, D28	0.004	1.2	1.15	0.6	4.5	23.5	115	436	2353	160	110	52	48	77	87
CESAR1, α=0.4, D28	< 0.1	1.3	1.19	0.3	4.7	24.1	111	315	2531	152	114	52	44	73	85
CESAR1, α=0.6, D10	< 0.1	1.1	1.07	< 0.1	2.1	11.7	78	161	914	296	107	43	27	45	52
CESAR1, α=0.6, D19	< 0.1	1.2	1.38	0.5	5.0	20.1	170	253	2131	161	86	43	33	63	72
CESAR1, α=0.6, D28	0.005	1.3	1.27	0.3	3.9	16.9	96	337	1867	131	85	42	26	52	63
CESAR1, α=0.6, D28	0.006	0.8		0.9	4.4		250	304		122	79		38	55	

Table S 5: Measured concentrations in thermally degraded AMP 3M, PZ 1.5M and CESAR1 in mg/kg, table 5 of 8.

	TMOX			GAc			FAc			AAc			PAc		
	120°C	135°C	150°C	120°C	135°C	150°C	120°C	135°C	150°C	120°C	135°C	150°C	120°C	135°C	150°C
AMP 3M, α=0.4, D28	743	1235	850	2.7	< 1	1.0	55	168	130	40	119	113	3.2	4.5	6.1
AMP 3M, α=0.4, D28	739	1218	906	1.6	< 1	1.5	69	78	260	28	47	148	3.7	3.6	5.8
PZ 1.5M, α=0.4, D28	< 0.1	< 1	< 1	17	17.3	15.8	37	126	344	17	20	26	< 1	2.2	1.9
PZ 1.5M, α=0.4, D28	< 0.1	< 1	< 1	18	19.4	14.9	52	81	240	12	27	23	< 1	1.5	3.7
CESAR1, α=0.1, D10	114	408	467	8.0	13.1	12.8	< 10	49	96	12	26	18	3.1	4.3	3.1
CESAR1, α=0.1, D19	205	605	464	9.0	11.9	13.6	14	58	167	27	27	39	3.8	3.6	6.0
CESAR1, α=0.1, D28	263	631	436	10	10.0	14.2	< 10	67	237	14	46	55	3.6	5.1	4.5
CESAR1, α=0.1, D28	229	598	435	12	13.1	16.1	12	64	238	16	39	72	3.4	4.1	5.2
CESAR1, α=0.4, D10	390	982	739	4.2	2.9	4.6	11	60	274	14	34	39	2.5	3.2	3.0
CESAR1, α=0.4, D19	568	997	660	3.2	4.8	< 1	18	82	611	13	32	< 10	2.8	2.9	7.8
CESAR1, α=0.4, D28	641	1085	626	10	5.3	< 1	41	118	1288	23	54	< 10	3.0	3.8	7.6
CESAR1, α=0.4, D28	650	896	646	4.7	5.4	< 1	18	150	1248	11	52	62	2.2	3.7	7.2
CESAR1, α=0.6, D10	425	976	688	2.5	2.7	< 1	< 10	127	403	11	32	< 10	1.7	3.6	< 1
CESAR1, α=0.6, D19	600	982	673	3.7	4.7	< 1	54	194	1245	15	47	< 10	2.5	3.9	< 1
CESAR1, α=0.6, D28	627	1076	656	2.4	2.7	< 1	42	216	789	20	53	65	3.7	4.0	7.8
CESAR1, α=0.6, D28	643	1001		3.3	6.6	< 1	78	196		24	47		2.8	3.3	

Table S 6: Measured concentrations in thermally degraded AMP 3M, PZ 1.5M and CESAR1 in mg/kg, table 6 of 8.

	LAc			iBAc			F-AMP			OPZ			DFP		
	120°C	135°C	150°C	120°C	135°C	150°C	120°C	135°C	150°C	120°C	135°C	150°C	120°C	135°C	150°C
AMP 3M, α=0.4, D28	< 10	< 10	< 10	< 1	2.0	10.4	18	33	32.7	< 1	< 1	< 1	< 1	< 1	< 1
AMP 3M, α=0.4, D28	< 10	< 10	< 10	3	1.2	10.4	23.4	24	59.9	< 1	< 1	< 1	< 1	< 1	< 1
PZ 1.5M, α=0.4, D28	< 10	< 10	< 10	3	< 1	< 1	< 1	< 1	< 1	62.3	80	114	< 1	< 1	< 1
PZ 1.5M, α=0.4, D28	< 10	23.5	< 10	< 1	< 1	< 1	< 1	< 1	< 1	51.9	76	95	< 1	< 1	< 1
CESAR1, α=0.1, D10	< 10	< 10	< 10	< 1	< 1	1.7	1.1	< 1	< 1	45.9	120	62	< 1	< 1	< 1
CESAR1, α=0.1, D19	< 10	17.5	< 10	61	< 1	< 1	2.0	1.0	1.7	57.2	93	53	< 1	< 1	< 1
CESAR1, α=0.1, D28	< 10	13.7	< 10	< 1	1.3	2.9	2.5	1.4	1.9	48.7	69	51	< 1	< 1	< 1
CESAR1, α=0.1, D28	< 10	< 10	< 10	79	< 1	1.3	2.0	1.4	2.6	58.3	74	55	< 1	< 1	< 1
CESAR1, α=0.4, D10	< 10	< 10	< 10	< 1	< 1	19.5	2.8	2.8	7.3	34.6	56	50	< 1	< 1	< 1
CESAR1, α=0.4, D19	< 10	< 10	< 10	< 1	< 1	12.2	3.9	4.2	14.0	17.4	53	34	< 1	< 1	< 1
CESAR1, α=0.4, D28	< 10	15.4	< 10	1	1.4	14.6	3.4	4.7	8.0	20.0	51	21	< 1	< 1	< 1
CESAR1, α=0.4, D28	< 10	< 10	< 10	< 1	1.3	15.7	3.3	5.8	6.8	16.3	52	24	< 1	< 1	< 1
CESAR1, α=0.6, D10	< 10	< 10	< 10	< 1	87.2	7.6	1.5	3.6	10.7	20.0	19	20	< 1	< 1	< 1
CESAR1, α=0.6, D19	< 10	13.1	< 10	< 1	80.8	18.4	3.3	8.0	12.8	14.5	21	22	< 1	< 1	< 1
CESAR1, α=0.6, D28	< 10	11.1	< 10	< 1	3.9	21.9	3.4	7.4	11.6	9.8	20	22	5.0	< 1	< 1
CESAR1, α=0.6, D28	< 10	< 10	< 10	< 1	4.3		4.6	8.3		11.2	14		2.5	< 1	

Table S 7: Measured concentrations in thermally degraded AMP 3M, PZ 1.5M and CESAR1, table 7 of 8.

	AIBA [mg/kg]			Formaldehyde [mg/kg]			Acetaldehyde [mg/kg]			AMP [g/kg]			PZ [g/kg]			TN [mg/kg]		
	120°C	135°C	150°C	120°C	135°C	150°C	120°C	135°C	150°C	120°C	135°C	150°C	120°C	135°C	150°C	120°C	135°C	150°C
AMP 3M, α=0.4, D28	2.4	17.0	14.9	13	44	62	< 10	8	6.2	233	< 0.001	181	215	< 0.1	0.01	38520	39943	41785
AMP 3M, α=0.4, D28	2.0	11.3	36.6	13	28	106	< 10	4	13	230	< 0.001	185	210	< 0.1	0.004	45722	40363	39946
PZ 1.5M, α=0.4, D28	< 1	< 5	< 1	6	9	45	< 10	3	2.3	< 0.1	117	0.2	< 0.1	117	117	37522	41005	39888
PZ 1.5M, α=0.4, D28	< 1	< 5	< 1	5	11	43	< 10	5	3.2	< 0.1	118	0.1	< 0.1	117	116	38011	42371	39988
CESAR1, α=0.1, D10	< 1	< 5	5.3	15	35	126	< 10	6	2.9	253	124	228	247	120	114	78536	81290	78543
CESAR1, α=0.1, D19	< 1	4.8	7.1	22	46	105	< 10	6	4.3	253	123	231	245	118	111	79867	81154	79472
CESAR1, α=0.1, D28	< 1	5.3	8.8	12	72	173	< 10	7	5.3	254	123	220	248	118	99	80036	82436	79237
CESAR1, α=0.1, D28	< 1	5.5	7.9	14	66	127	< 10	6	5.6	258	118	223	248	120	104	79191	81275	78536
CESAR1, α=0.4, D10	1.0	5.5	6.9	30	71	225	< 10	5	4.1	231	111	180	218	109	90	73490	77435	72220
CESAR1, α=0.4, D19	1.3	5.5	8.4	44	48	263	< 10	7	7.1	225	113	141	209	102	71	73868	75040	67796
CESAR1, α=0.4, D28	1.2	5.3	14.6	34	67	281	< 10	5	12.8	221	113	135	196	99	68	73332	75017	71902
CESAR1, α=0.4, D28	1.0	5.2	11.2	29	74	258	< 10	5	10.2	232	112	119	197	97	61	74027	75129	73380
CESAR1, α=0.6, D10	1.2	5.3	< 1	16	27	125	< 10	5	7.8	215	109	140	189	103	76	70448	72246	63936
CESAR1, α=0.6, D19	1.2	6.3	11.0	21	36	209	< 10	5	12.8	208	107	147	177	95	79	73799	72077	69379
CESAR1, α=0.6, D28	1.1	4.9	9.8	13	22	170	< 10	3	9.5	205	106	119	168	88	65	69059	70900	65979
CESAR1, α=0.6, D28	1.5	5.7		13	28		< 10	6		205	106		171	88		69863	70504	

Table S 8: Measured concentrations in thermally degraded AMP 3M, PZ 1.5M and CESAR1 in mg/kg, table 8 of 8.

	MA			EA			PA			DMA			EMA		
	120°C	135°C	150°C	120°C	135°C	150°C	120°C	135°C	150°C	120°C	135°C	150°C	120°C	135°C	150°C
AMP 3M, α=0.4, D28	3.0	2.9	2.6	0.4	0.4	0.7	1.3	1.2	1.0	0.5	< 0.1	0.2	< 0.1	< 0.1	0.13
AMP 3M, α=0.4, D28	3.3	3.6	3.2	0.3	0.4	0.6	1.3	1.1	1.0	0.2	< 0.1	0.3	< 0.1	< 0.1	0.15
PZ 1.5M, α=0.4, D28	0.2	1.2	1.7	0.1	0.2	0.8	< 0.1	< 0.1	< 0,1	< 0.1	< 0.1	0.1	< 0.1	< 0.1	< 0.1
PZ 1.5M, α=0.4, D28	< 0.1	0.3	0.5	< 0.1	0.8	0.4	< 0.1	< 0.1	< 0,1	0.2	< 0.1	< 0,1	< 0.1	< 0.1	< 0.1
CESAR1, α=0.1, D10	0.4	0.5	1.1	0.2	0.2	0.3	1.0	1.1	1.2	< 0.1	< 0.1	< 0,1	< 0.1	< 0.1	< 0.1
CESAR1, α=0.1, D19	1.0	1.2	2.4	0.4	0.2	0.3	1.2	1.1	1.3	0.2	< 0.1	0.2	< 0.1	< 0.1	< 0.1
CESAR1, α=0.1, D28	0.7	1.0	4.9	0.3	0.7	1.1	1.3	1.3	1.3	< 0.1	< 0.1	0.2	< 0.1	< 0.1	0.24
CESAR1, α=0.1, D28	0.5	1.1	4.2	0.2	0.2	0.4	1.1	1.0	1.2	< 0.1	< 0.1	0.2	< 0.1	< 0.1	< 0.1
CESAR1, α=0.4, D10	0.6	0.5	1.7	0.3	0.4	0.7	1.1	1.2	1.2	0.3	< 0.1	0.1	< 0.1	< 0.1	0.12
CESAR1, α=0.4, D19	3.5	2.6	3.0	0.4	0.5	< 0.1	1.2	1.1	1.2	0.3	< 0.1	< 0.1	< 0.1	< 0.1	< 0.1
CESAR1, α=0.4, D28	0.4	2.7	6.3	4.2	0.5	< 0.1	1.1	1.1	1.3	< 0.1	< 0.1	0.8	< 0.1	0.13	< 0.1
CESAR1, α=0.4, D28	0.8	3.0	5.3	0.4	0.6	< 0.1	1.1	1.1	1.4	< 0.1	< 0.1	< 0.1	< 0.1	0.29	< 0.1
CESAR1, α=0.6, D10	1.7	0.7	1.8	0.2	0.4	< 0.1	1.0	1.1	1.1	0.1	< 0.1	< 0.1	< 0.1	< 0.1	< 0.1
CESAR1, α=0.6, D19	1.1	1.5	3.5	0.3	0.8	< 0.1	1.0	1.0	1.3	0.2	< 0.1	< 0.1	< 0.1	0.57	< 0.1
CESAR1, α=0.6, D28	0.2	1.1	3.7	0.9	0.5	< 0.1	1.2	1.0	1.2	< 0.1	< 0.1	< 0.1	< 0.1	< 0.1	< 0.1
CESAR1, α=0.6, D28	3.5	1.3		0.5	0.3		1.2	1.0		0.3	< 0.1		< 0.1	< 0.1	

Table S 9: Total mass of each acid wash solution after oxidative degradation of the three solvents.

	Acid wash 1 (g)	Acid wash 2 (g)
**AMP 3M**	216.08	115.47
**PZ 1.5M**	129.72	134.98
**CESAR1**	228.43	121.73

Table S 10: Measured concentrations of specific compounds in acid washes from oxidative degradation experiments with AMP, PZ and CESAR1.

Experiment	Acid wash bottle	AMP	PZ	DAEP	AEAEPZ	PEP	AMPAMP	HMeGly	AEAEPZ urea	MAMP	DM-PZEA	F-AMP	DMOZD	DM-HTBI	AMP urea
		mg/kg													
AMP 3M	1	68.9	1.24	< 0.01	0.014	0.003	0.003	0.076	< 0.001	0.53	< 0.001	< 0,001	0.004	< 0.001	< 0.001
	2	36.5	0.74	< 0.01	< 0.001	< 0.001	0.019	0.087	< 0.001	0.34	< 0.001	< 0,001	0.003	< 0.001	< 0.001
PZ 1.5M	1	6.23	188.1	< 0.01	< 0.001	< 0.001	< 0.001	0.014	< 0.001	0.14	< 0.001	< 0,001	0.004	< 0.001	< 0.001
	2	< 0.01	2.63	< 0.01	< 0.001	< 0.001	< 0.001	0.020	< 0.001	< 0.001	< 0.001	< 0,001	0.005	< 0.001	< 0.001
CESAR1	1	262.4	93.5	< 0.01	< 0.001	< 0.001	< 0.001	0.262	< 0.001	0.93	0.004	0.004	0.005	< 0.001	< 0.001
	2	0.7	3.5	< 0.01	< 0.001	< 0.001	< 0.001	0.188	< 0.001	0.004	< 0.001	0.041	0.003	< 0.001	< 0.001

Table S 11: Measured concentrations of specific compounds in acid washes from oxidative degradation experiments with AMP, PZ and CESAR1.

Experiment	Acid wash bottle	HTBI	TMOX	AEAAC	OPZ	AEHA	AIBA	FPZ	HMTA	DFP	HEP	MPZ	DMP	AMPP	EPZ	AAEA
		mg/kg														
AMP 3M	1	< 0.01	< 0.01	0.045	< 0.01	< 0.001	< 0,005	0.25	< 0,001	< 0,001	< 0.001	0.041	< 0.001	< 0.001	0.010	0.015
	2	< 0.01	< 0.01	0.033	< 0.01	< 0.001	< 0,005	0.018	< 0,001	< 0,001	< 0.001	0.021	< 0.001	< 0.001	0.007	< 0.005
PZ 1.5M	1	< 0.01	< 0.01	0.012	< 0.01	< 0.001	< 0,005	0.010	< 0,001	< 0,001	< 0.001	0.036	< 0.001	< 0.001	0.021	< 0.005
	2	< 0.01	< 0.01	0.087	< 0.01	< 0.001	< 0,005	< 0.01	< 0,001	< 0,001	< 0.001	< 0.001	< 0.001	< 0.001	< 0.001	< 0.005
CESAR1	1	< 0.01	< 0.01	< 0,01	< 0.01	0.005	1.77	0.564	< 0,001	0.009	< 0.001	0.037	< 0.001	< 0.001	0.002	< 0.005
	2	< 0.01	< 0.01	< 0,01	< 0.01	< 0.001	0.12	< 0.01	< 0,001	< 0,001	< 0.001	< 0.001	< 0.001	< 0.001	< 0.001	< 0.005

Table S 12: Measured concentrations of specific compounds in acid washes from oxidative degradation experiments with AMP, PZ and CESAR1.

Experiment	Acid wash bottle	EDA	Formaldehyde	Acetaldehyde	Acetone	NH_3	MA	EA	PA	DMA	EMA	DEA	DPA
		mg/kg											
AMP 3M	1	14.7	5.04	< 0.02	< 0.05	14.9	0.0879	0.0139	0.0848	0.0024	< 0.0005	< 0.0005	0.0012
	2	5.6	1.15	< 0.02	< 0.05	3.3	0.0623	0.0066	0.0010	0.0031	< 0.0005	< 0.0005	< 0.0005
PZ 1.5M	1	19.8	0.44	< 0.02	< 0.05	69.4	0.0526	0.0063	0.0046	0.0026	< 0.0005	< 0.0005	< 0.0005
	2	0.5	0.36	< 0.02	< 0.05	0.8	0.0044	< 0.0005	< 0.0005	0.0026	< 0.0005	< 0.0005	< 0.0005
CESAR1	1	8.7	17.25	2.03	194.6	0.1574	0.1772	0.1993	0.0203	0.0184	< 0.0005	0.0118	0.1574
	2	0.4	7.91	1.33	134.9	0.0064	< 0.0005	0.0026	< 0.0005	< 0.0005	< 0.0005	< 0.0005	0.0064

Table S 13: Measured concentrations in the oxidative degradation experiments of 3M AMP, 1.5M PZ and CESAR1, table 1 of 4.

	Day	Alkalinity	TN	CO_2	AMP	PZ	GAc	LAc	FAc	HBAc	AAc	PAc	iBAc	BAc	DAEP	AEAEPZ	PEP	AMPAMP
		mol/kg	mg/kg	mol/kg	g/kg	g/kg	mg/kg											
AMP 3M	0	2.81	38451	0.82	248	0.002	< 1	< 10	< 10	< 1	< 10	< 1	< 1	< 1	< 1	< 1	< 0.1	2.0
	7	3.80	51089	0.85	330	0.004	< 1	< 10	23.0	< 1	< 10	< 1	< 1	< 1	< 1	< 1	< 0.1	2.8
	14	3.73	49467	0.84	320	0.004	< 1	< 10	15.5	< 1	< 10	< 1	< 1	< 1	< 1	< 1	< 0.1	3.0
	21	3.47	44807	0.82	298	0.004	< 1	< 10	18.5	< 1	< 10	< 1	< 1	< 1	< 1	< 1	< 0.1	2.9
	28	3.26	44251	0.82	289	0.004	< 1	< 10	21.0	< 1	< 10	< 1	< 1	< 1	< 1	< 1	< 0.1	3.0
	35	3.61	48769	0.82	318	0.004	< 1	< 10	13.1	< 1	< 10	< 1	< 1	< 1	< 1	< 1	< 0.1	3.0
PZ 1.5M	0	2.94	40022	0.82	0.04	126.1	< 1	< 10	< 10	< 1	< 10	< 1	< 1	< 1	< 1	< 1	< 0.1	< 0.1
	7	2.86	39477	0.85	0.09	117.3	2.9	< 10	< 10	< 1	< 10	< 1	< 1	< 1	< 1	< 1	< 0.1	< 0.1
	14	2.83	38604	0.85	0.09	120.1	4.0	< 10	13.9	< 1	< 10	< 1	< 1	< 1	< 1	< 1	< 0.1	< 0.1
	21	2.83	38977	0.84	0.09	120.6	4.5	< 10	15.8	< 1	< 10	< 1	< 1	< 1	< 1	< 1	< 0.1	< 0.1
	28	2.77	38413	0.83	0.09	118.6	5.5	< 10	< 10	< 1	< 10	< 1	< 1	< 1	< 1	< 1	< 0.1	< 0.1
	35	2.78	37344	0.82	0.10	117.6	5.4	< 10	16.8	< 1	< 10	< 1	< 1	< 1	< 1	< 1	< 0.1	< 0.1
	42	2.71	36030	0.82	0.10	116.5	5.6	< 10	12.8	< 1	< 10	< 1	< 1	< 1	< 1	< 1	< 0.1	< 0.1
CESAR1	0	5.44	75870	2.11	323	150	< 1	< 10	61	< 1	<10	< 1	< 1	< 1	< 1	< 0,1	< 0,1	6.01
	7	5.42	77970	1.55	248	115	1.2	< 10	307	< 1	< 10	< 1	< 1	< 1	< 1	< 0,1	< 0,1	0.27
	14	5.32	78856	1.50	235	110	3.6	< 10	1297	< 1	19	1.7	< 1	< 1	< 1	< 0,1	< 0,1	0.11
	21	5.11	75375	1.42	229	105	6.1	< 10	1931	< 1	43	2.4	< 1	< 1	< 1	< 0,1	< 0,1	0.14
	28	4.99	71922	1.36	224	102	8.7	< 10	2813	< 1	59	2.7	< 1	< 1	< 1	< 0,1	< 0,1	0.24
	35	4.85	72989	1.37	221	98.1	10.9	< 10	3521	< 1	83	3.7	< 1	< 1	< 1	< 0,1	< 0,1	0.33
	42	5.91	89805	1.69	266	117.2	12.6	20.7	4716	< 1	122	4.4	< 1	< 1	< 1	< 0,1	< 0,1	0.56

Table S 14: Measured concentrations in the oxidative degradation experiments of 3M AMP, 1.5M PZ and CESAR1, table 2 of 4.

	Day	HMeGly	AEI	AEAEPZ Urea	MAMP	DM-PZAE	F-AMP	DMOZD	DM-HTBI	AMP Urea	HTBI	TMOX	AEAAC	OPZ	AEHA
		mg/kg													
AMP 3M	0	0.6	< 0.1	< 0.1	724	< 0.1	16.8	0.8	< 0.1	< 0.1	< 0.1	< 0.1	1.5	0.8	< 0.1
	7	1.3	< 0.1	< 0.1	998	< 0.1	3.2	12.7	< 0.1	< 0.1	< 0.1	1.1	2.5	0.8	< 0.1
	14	1.5	< 0.1	< 0.1	985	< 0.1	1.0	33.7	< 0.1	< 0.1	< 0.1	3.1	2.5	0.9	< 0.1
	21	1.7	< 0.1	< 0.1	926	< 0.1	0.5	40.4	< 0.1	< 0.1	< 0.1	3.8	2.2	0.9	< 0.1
	28	1.7	< 0.1	< 0.1	895	< 0.1	0.3	49.1	< 0.1	< 0.1	< 0.1	4.7	2.1	1.0	< 0.1
	35	1.7	< 0.1	< 0.1	971	< 0.1	0.4	63.5	< 0.1	< 0.1	< 0.1	6.1	2.2	0.9	< 0.1
PZ 1.5M	0	< 1	< 0.1	< 0.1	0.2	< 0.1	< 1	< 1	< 0.1	< 0.1	< 0.1	< 0.1	6.5	60	< 0.1
	7	< 1	< 0.1	< 0.1	0.4	< 0.1	< 1	< 1	< 0.1	< 0.1	< 0.1	< 0.1	63	107	0.5
	14	< 1	< 0.1	< 0.1	0.4	< 0.1	< 1	< 1	< 0.1	< 0.1	< 0.1	< 0.1	72	94	0.5
	21	< 1	< 0.1	< 0.1	0.4	< 0.1	< 1	< 1	< 0.1	< 0.1	< 0.1	< 0.1	72	91	0.5
	28	< 1	< 0.1	< 0.1	0.5	< 0.1	< 1	< 1	< 0.1	< 0.1	< 0.1	< 0.1	71	93	0.4
	35	< 1	< 0.1	< 0.1	0.5	< 0.1	< 1	< 1	< 0.1	< 0.1	< 0.1	< 0.1	69	94	0.4
	42	< 1	< 0.1	< 0.1	0.5	< 0.1	< 1	< 1	< 0.1	< 0.1	< 0.1	< 0.1	67	95	0.4
CESAR1	0	171.7	< 0,1	< 0,1	1576	< 0,1	3.6	1.4	< 0,1	< 0,1	< 1	< 1	5.3	25.7	< 0,1
	7	2252	< 0,1	< 0,1	1005	< 0,1	11	12	0.33	< 0,1	< 1	< 1	197	548	1.8
	14	3608	< 0,1	< 0,1	956	0.47	22	40	0.63	0.2	< 1	< 1	396	1086	4.7
	21	4487	< 0,1	< 0,1	898	1.16	27	63	0.51	0.3	< 1	< 1	491	1463	7.7
	28	5031	< 0,1	< 0,1	886	1.84	33	86	0.40	0.3	< 1	< 1	584	1656	11
	35	5668	< 0,1	< 0,1	887	2.11	42	112	0.38	0.4	< 1	< 1	569	1906	13.5
	42	7714	< 0,1	< 0,1	1071	4.26	166	171	0.51	0.5	< 1	< 1	674	3341	22.3

Table S 15: Measured concentrations in the oxidative degradation experiments of 3M AMP, 1.5M PZ and CESAR1, table 3 of 4.

	Day	AIBA	FPZ	DFP	HEP	MPZ	DMP	AMPP	EPZ	AAEA	EDA	MNPZ	NMAMP	AMP-NO_2	PZ-NO_2
					mg/kg									µg/kg	
AMP 3M	0	< 1	< 0.1	< 1	< 0.1	< 0.1	< 1	< 0.1	< 0.1	< 0.1	31	< 0.1	< 0.1	< 10	n.a.
	7	< 1	< 0.1	< 1	< 0.1	< 0.1	< 1	< 0.1	< 0.1	< 0.1	59	< 0.1	< 0.1	< 10	n.a.
	14	< 1	< 0.1	< 1	< 0.1	< 0.1	< 1	< 0.1	< 0.1	< 0.1	65	< 0.1	< 0.1	< 10	n.a.
	21	< 1	< 0.1	< 1	< 0.1	< 0.1	< 1	< 0.1	< 0.1	< 0.1	56	< 0.1	< 0.1	< 10	n.a.
	28	< 1	< 0.1	< 1	< 0.1	< 0.1	< 1	< 0.1	< 0.1	< 0.1	50	< 0.1	< 0.1	< 10	n.a.
	35	< 1	< 0.1	< 1	< 0.1	< 0.1	< 1	< 0.1	< 0.1	< 0.1	56	< 0.1	< 0.1	< 10	n.a.
PZ 1.5M	0	< 1	9.0	< 1	< 0.1	< 0.1	< 1	< 0.1	0.28	< 0.1	14	< 1	< 0.1	< 10	< 1000
	7	< 1	9.9	< 1	< 0.1	< 0.1	< 1	< 0.1	0.33	< 0.1	77	< 1	< 0.1	< 10	< 1000
	14	< 1	10.0	< 1	< 0.1	< 0.1	< 1	< 0.1	0.3	< 0.1	99	< 1	< 0.1	< 10	< 1000
	21	< 1	10.1	< 1	< 0.1	< 0.1	< 1	< 0.1	0.28	< 0.1	110	< 1	< 0.1	< 10	< 1000
	28	< 1	13.4	< 1	< 0.1	< 0.1	< 1	< 0.1	0.28	< 0.1	118	< 1	< 0.1	< 10	< 1000
	35	< 1	11.5	< 1	< 0.1	< 0.1	< 1	< 0.1	0.29	< 0.1	130	< 1	< 0.1	< 10	< 1000
	42	< 1	11.4	< 1	< 0.1	< 0.1	< 1	< 0.1	0.28	< 0.1	142	< 1	< 0.1	< 10	< 1000
CESAR1	0	< 5	70.1	< 1	< 0,1	< 0,1	< 0,1	< 0,1	0.55	< 0,5	65.3	< 1	< 0,1	< 10	< 1000
	7	5.3	1056	< 1	< 0,1	2.1	< 0,1	< 0,1	0.36	0.6	913	20	< 0,1	44	2433
	14	6.4	2626	< 1	0.1	7.9	< 0,1	0.16	0.34	3.0	1810	85	0.15	111	6478
	21	8.0	3863	< 1	0.2	15	< 0,1	0.26	0.32	6.3	2273	199	0.32	147	9161
	28	16.1	4934	2.1	0.4	24	< 0,1	0.31	0.33	11.6	2730	313	0.51	215	10974
	35	12.8	6033	3.3	0.5	35	< 0,1	0.33	0.31	17.3	2947	467	0.73	277	14722
	42	21.4	10653	10.1	1.0	59	< 0,1	0.46	0.40	33.4	4085	7556	1.20	371	21489

Table S 16: Measured concentrations in the oxidative degradation experiments of 3M AMP, 1.5M PZ and CESAR1, table 4 of 4.

	Day	Formaldehyde	Acetaldehyde	Acetone	NH_3	MA	EA	PA	DMA	EMA	DEA
		mg/kg									
AMP 3M	0	< 10	< 10	< 50	14.0	1.5	< 0.1	< 0.1	< 0.1	< 0.1	< 0.5
	7	< 10	< 10	< 50	13.1	2.1	< 0.1	< 0.1	< 0.1	< 0.1	< 0.5
	14	< 10	< 10	< 50	10.9	1.8	< 0.1	< 0.1	< 0.1	< 0.1	< 0.5
	21	< 10	< 10	< 50	28.2	1.7	< 0.1	< 0.1	< 0.1	< 0.1	< 0.5
	28	< 10	< 10	< 50	10.7	1.9	< 0.1	< 0.1	< 0.1	< 0.1	< 0.5
	35	< 10	< 10	< 50	< 10	1.9	< 0.1	< 0.1	< 0.1	< 0.1	< 0.5
PZ 1.5M	0	2.3	< 10	< 50	< 10	< 0.1	< 0.1	< 0.1	< 0.1	< 0.1	< 0.1
	7	5.5	< 10	< 50	< 10	< 0.1	< 0.1	< 0.1	< 0.1	< 0.1	< 0.1
	14	5.9	< 10	< 50	< 10	< 0.1	< 0.1	< 0.1	< 0.1	< 0.1	< 0.1
	21	6.4	< 10	< 50	< 10	< 0.1	< 0.1	< 0.1	< 0.1	< 0.1	< 0.1
	28	6.9	< 10	< 50	< 10	< 0.1	< 0.1	< 0.1	< 0.1	< 0.1	< 0.1
	35	7.4	< 10	< 50	< 10	< 0.1	< 0.1	< 0.1	< 0.1	< 0.1	< 0.1
	42	7.8	< 10	< 50	< 10	< 0.1	< 0.1	< 0.1	< 0.1	< 0.1	< 0.1
CESAR1	0	166	4.95	163	19	< 0.1	< 0.1	0.6	< 0.1	< 0.1	< 0.1
	7	964	2.29	436	121	< 0.1	< 0.1	0.7	< 0.1	< 0.1	< 0.1
	14	1180	2.44	338	130	1.8	1.0	0.7	< 0.1	< 0.1	< 0.1
	21	1164	3.60	279	119	2.5	1.6	0.8	< 0.1	< 0.1	< 0.1
	28	1064	4.10	185	117	3.3	2.4	0.9	< 0.1	< 0.1	< 0.1
	35	965	5.13	140	111	3.7	3.3	0.7	< 0.1	< 0.1	< 0.1
	42	1124	2.59	83	132	3.4	2.3	0.8	< 0.1	< 0.1	< 0.1
